# Influence of Surface Texture in Additively Manufactured Biocompatible Materials and Triboelectric Behavior

**DOI:** 10.3390/ma18143366

**Published:** 2025-07-17

**Authors:** Patricia Isabela Brăileanu, Nicoleta Elisabeta Pascu

**Affiliations:** Department of Robotics and Manufacturing Systems, Faculty of Industrial Engineering and Robotics, National University of Science and Technology POLITEHNICA Bucharest, 060042 Bucharest, Romania; nicoleta.pascu@upb.ro

**Keywords:** triboelectric nanogenerators, biocompatible materials, additive manufacturing, biomedical applications, surface texture, surface modification, polymers

## Abstract

This study analyzes the recent scientific literature on advanced biocompatible materials for triboelectric nanogenerators (TENGs) in biomedical applications. Focusing on materials like synthetic polymers, carbon-based derivatives, and advanced hybrids, the study interprets findings regarding their triboelectric properties and performance influenced by surface texture and additive manufacturing techniques. Major findings reveal that precise control over surface morphology, enabled by additive manufacturing (AM) is promising for optimizing transferred charge density and maximizing TENG efficiency. The analysis highlights the relevance of these material systems and fabrication strategies for developing self-powered wearable and implantable biomedical devices through enabling biocompatible energy-harvesting components that can operate autonomously without external power, underscoring the need for stringent biocompatibility and performance stability. This work synthesizes current progress, identifying critical material and process design parameters for advancing the field of biocompatible TENGs.

## 1. Introduction

Additive manufacturing (AM) techniques significantly influence the design and performance of TENGs through their ability to fabricate custom and functional structures using advanced biomaterials that allow surface texture optimization at both the micro- and nanoscale, aiming to improve specific performance characteristics, such as electrical or mechanical properties [1]. For instance, a newly designed 3D-printed biomimetic-villus structure-based TENG (BV-TENG) has been demonstrated using polytetrafluoroethylene (PTFE) powder and acrylonitrile butadiene styrene (ABS) as active triboelectric materials, showcasing AM’s capability for specific TENG geometries, by H.J. Yoon et al. [2].

**Figure 1:**
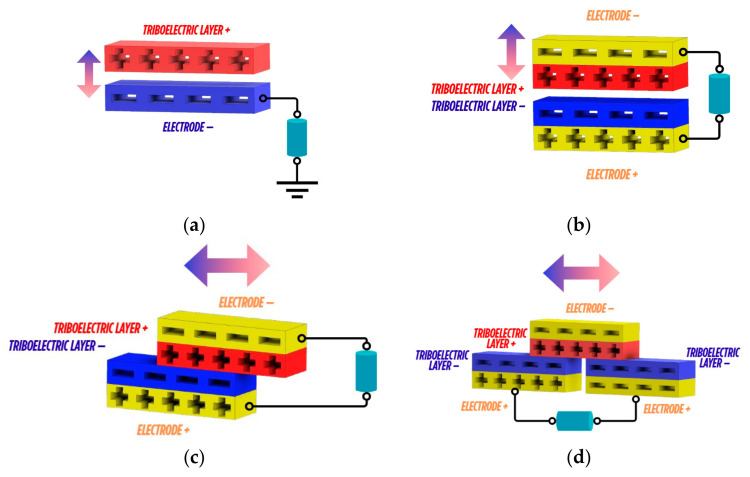
TENGs operating modes: (**a**) single electrode; (**b**) contact separation; (**c**) linear sliding; (**d**) freestanding.

The working principle of TENGs varies according to their intended application, and they can be classified into four distinct modes: single electrode (Figure 1a), contact separation (Figure 1b), linear sliding (Figure 1c), and freestanding triboelectric layer configurations (Figure 1d). TENGs represent a promising solution to address the energy crisis by converting ambient mechanical energy into electrical signals [3,4,5], and their ability to harvest low-frequency waste mechanical energy with high conversion efficiency positions them as a green and clean energy-harvesting technology [6,7,8].

The conductivity of different materials, including the ionic conductivity found in gel polymer electrolyte electrodes and the addition of carbon nanotubes and graphene, has a direct impact on the electrical output of TENG by improving charge transfer, current, and voltage [3,5,9,10,11,12,13,14,15]. The flexibility and stretchability, inherent in materials like polydimethylsiloxane (PDMS) or Ecoflex^®^, enable sustainable mechanical motion harvesting [9,16,17,18,19,20]. Moreover, dielectric permittivity is mandatory for materials with higher relative permittivity or those incorporating high-permittivity nanoparticles or ferroelectric polymers, resulting in greater charge density, higher capacitance, and consequently, amplified voltage and current outputs [11,15,21].

Integrating triboelectric technology through AM makes it possible to create medical devices tailored to patients’ anatomical landmarks, enabling adaptable and custom devices [22] for various biomedical applications. Although advanced biomaterials have differing electronic properties such as work function and electronic structure [23], the effectiveness of triboelectric charge generation critically depends on surface properties such as roughness, porosity, and charge affinity. A study by M. Kang et al. [24] demonstrated that this phenomenon can be significantly amplified through the generation of specific surface morphologies that are easily created via AM.

Surface modification, including texturing and patterning, is a fundamental approach to enhance triboelectric charges [10,20,25]. In the context of AM, thermal imprinting lithography has been specifically utilized to create microarchitectured PTFE surfaces, demonstrating enhanced electrical output through optimized imprinting parameters [26]. According to J.L. Armitage et al., freestanding layers polished to low surface roughness produced a significantly higher charge density compared to rougher surfaces [27]. This control over topography via layer-by-layer with 3D printing has proven highly reliable for TENGs with hierarchical designs, as shown by C. Qian et al.’s study, where output voltages were approximately 75% higher compared to similar devices made via conventional molding [25]. Due to recent research in this field, additive manufacturing opens a promising platform for optimizing surface textures to maximize triboelectric charge, enabling the generation of complex or custom geometries to create highly efficient energy-harvesting devices.

The literature lacks systematic studies on the tribological safety of textured surfaces under simulated physiological conditions. Friction, inherent in TENG operation, could increase the risk of infection and inflammation when TENGs are applied to body implants [28]. Also, the lack of mass production and facile fabrication techniques for stable nanostructure arrays, such as peptide-based materials, continues to hinder practical applications [29]. Moreover, the uneven dispersion of nanomaterials within composite friction layers remains a critical challenge, as aggregation can compromise TENG performance [15].

New perspectives regarding optimal biocompatible material selection for biomedical systems propose the use of advanced nanomaterials such as MXene and graphene [30] to enhance device efficiency. MXene, due to its high electronegativity and conductivity, was proposed by Q. Yi et al. and integrated into a 3D-printed, flexible triboelectric system, demonstrating effective energy conversion [31]. In the self-powered wearable electronics presented by X. Meng et al., graphene was introduced in the form of conductive composite paper [30], eliminating the need for traditional metal electrodes and enabling fully biodegradable, high-performance devices.

Although these advancements exist, several critical challenges remain in the development of biocompatible TENGs:

1. Optimization of surface texture for enhanced triboelectric performance;
2. Long-term stability under physiological conditions;
3. Scalability of manufacturing processes;
4. Integration with existing biomedical device technologies.

This review aims to address challenges by systematically analyzing the triboelectric properties of advanced biocompatible materials, evaluating the impact of AM techniques on surface texture optimization, identifying key material and process design parameters for biomedical TENGs, and offering an in-depth evaluation of current limitations and future research directions.

Through the synthesis of recent developments in materials science and AM technologies, this work seeks to establish a comprehensive framework for the design and implementation of biocompatible TENGs in next-generation medical devices.

## 2. Review Methodology

### 2.1. Search Strategy

This systematic review followed a structured approach to identify, select, and analyze the relevant literature:

The main databases used in this study include the following:

- Web of Science;
- Scopus;
- IEE Xplore;
- PubMed.

The keywords used to identify the relevant literature were as follows: (“triboelectric” or “TENG”) and (“biocompatible” or “implantable”) and (“additive manufacturing” or “3D printing”) and (“surface texture” or “morphology”). The timeframe of 2015–2025 was deliberately selected to capture recent developments in the field, except for a few earlier studies included for context or foundational relevance. A total of approximately 1500 articles were analyzed to identify and compare surface modification methods across various materials relevant to triboelectric applications.

Inclusion parameters for literature selection:

1. Studies reporting quantitative performance data;
2. Research on biocompatible materials;
3. Papers addressing surface texture modification;
4. Articles with clear experimental methodologies.

### 2.2. Analysis Framework

The analysis framework was structured around four key dimensions to ensure a comprehensive and systematic evaluation of the selected studies:

1. Material classification;
2. Surface modification techniques;
3. Performance metrics comparison;
4. Biomedical applications assessment.

## 3. Advanced Biocompatible Materials for Triboelectric Applications

Advanced biocompatible materials represent a group of materials that must simultaneously fulfill two essential conditions: biological compatibility in relation to the human body (biocompatibility) and advanced performance or technological functionality. In terms of biocompatibility, we generally refer to a material as biocompatible if it does not cause adverse reactions when in contact with human tissues or biological fluids (e.g., skin, blood, or various mucous membranes), meaning it is not toxic, irritating, or inflammatory; in short, it can be tolerated by the body both in the short and long term, depending on the operating environment.

Advanced performance refers to optimized physicochemical properties such as electrical conductivity (in the case of TENGs), elasticity, transparency, flexibility, thermal and chemical stability, as well as nano- or microscale structuring. These advanced materials are typically designed for specific applications such as triboelectricity, chemical sensing, or ionic transport.

**Figure 2:**
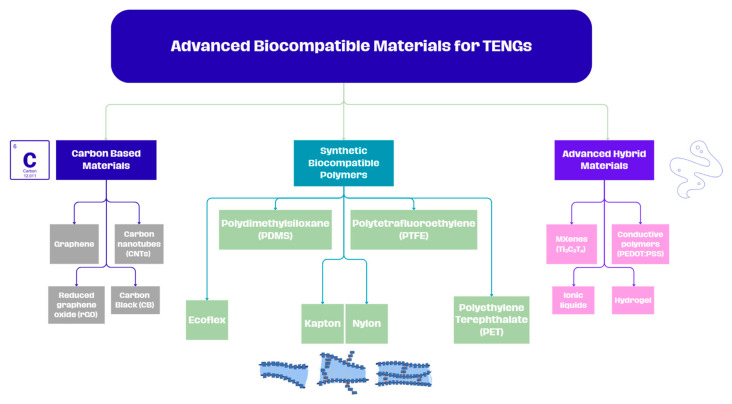
Advanced biocompatible materials used in TENGs and analyzed in this study.

The following section will analyze the most used advanced biocompatible materials in the construction of TENGs, which exhibit essential triboelectric properties for such applications. Figure 2 groups all the materials analyzed in this study into three main categories: synthetic biocompatible polymers, carbon-based materials, and the emerging class of advanced hybrid materials.

### 3.1. Synthetic Biocompatible Polymers

#### 3.1.1. Polydimethylsiloxane (PDMS)

PDMS has emerged as an exemplar triboelectric polymer and is frequently employed in the development of TENGs [18,32,33,34]. The triboelectric effect involves charge transfer between surfaces upon contact and separation, driven by mechanisms including electron, ion, and material transfer (bond cleavage) [33]. G. Wang et al.’s study on ZnSnO_3_ nanocube/PDMS composites for TENGs suggest that the charge generation mechanism involves bond breaking within the PDMS matrix [18]. Reactive oxygen radicals, potentially produced by the ZnSnO_3_, can attack methyl groups (Si-CH_3_) on the PDMS chains, substituting them with silanol groups (Si-OH) and leading to an increase in redundant dangling bonds, and consequently, surface charge density [18]. Therefore, in the studies by G. Wang et al.’s the effect was observed to increase the TENG output voltage and current up to a certain filler concentration (6 wt% ZnSnO_3_), after which the output decreased [18].

The surface properties of PDMS, including texture, roughness, and chemistry, significantly influence its triboelectric behavior [28,33]. Engineering the surface topography, such as increasing porosity and surface roughness, is a known strategy to enhance TENG performance, as shown by J. Li et al. [33]. For instance, in D. Choi et al.’s study, highly embossed surface (SE) PDMS patterns fabricated on fabric with the assistance of ZnO nanoframes demonstrated enhanced surface charge density due to enlarged contact areas, thereby boosting electrostatic induction [35]. Creating specific micro/nanotextures on PDMS surfaces is a research point of interest for achieving functional surfaces [36]. Z. Bingpu et al. reported PDMS surfaces with controlled roughness gradients, achieving average roughness values ranging from 2.6 ± 0.7 nm to 163.6 ± 11.7 nm [37].

Surface chemical modification is another crucial approach to tailor PDMS triboelectric properties. Various methods, like those used by N. Luo et al., S. Vlassov et al., and T. Artur et al., including plasma treatment (argon, CF_4_/O_2_, oxygen), base treatment, and irradiation, were explored [34,38,39]. Argon plasma treatment and base treatment have been reported by N. Luo et al. to enhance the output of PDMS based TENGs. Exposure to atomic oxygen (AO) has been demonstrated to alter the electrical properties of PDMS films by inducing the conversion of Si–C bonds into Si–O bonds, thereby increasing electron-donating groups and enhancing their positive charge characteristics [34]. Consequently, as mentioned, six hours of AO irradiation significantly increased the short-circuit current from 5 μA to 22 μA, and the output voltage from 160 V to 760 V in N. Luo et al.’s research for PTFE–PDMS TENG configuration [34]. Plasma treatment has also been used in conjunction with composite materials, such as CF_4_/O_2_ plasma treatment of PDMS/PTFE composites, to selectively etch PDMS and reveal hydrophobic PTFE particles, creating superhydrophobic surfaces with the potential for reduced drag, as shown by T. Artur et al. [39].

Surface treatments may also be designed to improve biocompatibility or prevent protein adsorption, as shown by J. Liu et al., which is critical for biomedical applications, by increasing hydrophilicity through methods like plasma treatment or grafting with polar groups or zwitterionic polymers [36]. UV-ozone pre-cleaning is also used by A. Jain et al. to reduce organic residues on the PDMS surface, which is important for subsequent processes [40].

Additive manufacturing techniques are increasingly employed to fabricate complex PDMS structures, opening avenues for integrating PDMS into custom wearable and biomedical devices, including TENGs. Direct ink writing is a common method for 3Dprinting PDMS, often requiring rheology optimization through blending the PDMS components of different viscosities or adding fillers like silica nanoparticles [41,42,43]. Porous structures created by removing a sacrificial component (e.g., dibutyl phthalate, DBP) can exhibit specific pore sizes, reported by R. Woo et al. to range from 0.8 μm to 7.5 μm with an average of 2.4 ± 1.5 μm [43]. Digital light processing (DLP) is another AM technique capable of printing PDMS-based materials, allowing for complex microarchitecture, high resolution, and good surface finishing, which is valuable for medical devices, wearable devices, and soft robotics [44]. Design templating, where a 3D-printed mold (e.g., water-soluble filament via fused deposition modeling, FDM) is used for casting PDMS, allows for the fabrication of complex microfluidic-like structures [45,46].

In the context of wearable and biomedical energy harvesting, PDMS offers significant advantages due to its inherent biocompatibility, flexibility, and stretchability [36,43,44,47,48,49,50]. Its low cost and ease of processing also facilitate prototyping and scaling [45,48,49,50,51]. The ability to modify its surface properties and create composites allows for tuning wettability, anti-fouling characteristics, and mechanical strength, which are essential for interaction with biological environments, as shown by J. Liu et al. and R. Ariati et al. [36,50]. However, the low intrinsic mechanical strength of pure PDMS must often be addressed through bulk modification or fillers [47,50]. Furthermore, while PDMS is biocompatible, the tribological safety of textured surfaces under simulated physiological conditions requires systematic study, as friction could potentially increase the risk of infection and inflammation on body implants, as shown by Y. Xi et al. [28]. Certain textures, like the micropillars from Y. Xi et al.’s research, may also face practical limitations regarding wear resistance in real applications [28]. Despite these challenges, the combination of PDMS’s basic properties, tunable surface characteristics (Table 1), and compatibility with AM techniques positions it as a promising material for developing advanced triboelectric energy harvesters integrated into biomedical and wearable technologies. PDMS additionally demonstrates strong tribonegative characteristics and is frequently integrated into TENG systems due to its low electrical resistance and compatibility with nanostructured composites [11,52].

#### 3.1.2. Polyfluoroethylene (PTFE)

Polytetrafluoroethylene, widely recognized by the trade name Teflon™ (DuPont, Wilmington, DE, USA), is a synthetic fluoropolymer composed exclusively of carbon and fluorine atoms [53,54,55]. It is frequently used in machine elements due to its favorable tribological properties such as low friction, chemical inertness, and high-temperature resistance [56]. PTFE biocompatibility significantly expands its application potential, supporting integration into medical devices and implants, including expanded PTFE (e-PTFE) for soft tissue engineering and biliary metal stents to control tumor ingrowth [54,55,57,58,59,60,61]. However, the inherent hydrophobicity and poor wettability of pristine PTFE can hinder applications requiring cell adhesion, such as biomedical implants, necessitating surface modifications [53,57].

PTFE holds a prominent position as one of the most efficient polymers for TENGs, largely due to its extremely strong electron affinity, placing it high on the tribonegative side of the triboelectric series [21,27,62,63]. This characteristic allows PTFE to effectively acquire and retain negative charge during contact electrification (A. Ciniero et al., G. Fatti et al.) [62,63]. It is also an excellent electret material, as shown in P. White et al.’s study, capable of quasi-permanently trapping the surface charge [21]. Consequently, PTFE is widely used as a triboelectric layer in TENG devices, and some examples we can find are in research performed by P. White et al., which investigated the energy-harvesting performance using materials such as PTFE derived from a triboelectric assembly, while R. Zheng et al. developed PTFE structures via 3D printing and integrated them into a TENG system [2,21,64].

Studies performed by J.L. Armitage et al. using sliding–freestanding triboelectric layer TENGs (F-TENGs) have shown that Teflon freestanding layers initially accumulate charge at a faster rate than layers composed of Delrin (Polyoxymethylene, POM) [27]. Therefore, based on the results reported by J.L. Armitage et al., the charge accumulation for an F-TENG contact exhibits both exponential and logarithmic trends, dependent on surface compositions and roughness parameters [27]. PTFE’s outstanding capacity for electron capture is strongly influenced by the electronic behavior of its inherent structural defects (A. Ciniero et al., G. Fatti et al.) [62,63]. A. Ciniero et al. and G. Fatti et al. have shown that defects like fluorine vacancies or bent chains, which can form under the high temperatures and stresses of frictional processes, can generate trap states that enable the surface to acquire and retain negative charge, energetically stabilizing the material upon negative charging [62,63]. Table 2 provides an overview of AM strategies and surface modifications applied to PTFE-based TENGs, highlighting their functional performance.

The cumulative triboelectric charging effect on the PTFE surface can significantly amplify the output voltage of TENG-based sensors, such as self-powered particle sensors (PS-TENG), as reported by L. Wang et al. [65]. As a result, these sensors utilize triboelectrification between particles and the PTFE surface to characterize particles based on detecting induced outputs [65]. In L. Wang et al.’s example, PTFE tubes were selected as a triboelectric surface due to their ability to maintain a large contact area with spherical particles [65]. The triboelectric performance of PTFE can be significantly influenced by its surface characteristics. Increasing surface roughness and surface area may generally favor enhanced charge transfer and TENG output [26].

The conventional processing of PTFE, such as injection or extrusion, is challenging due to its high melt viscosity and poor flowability [54,55,58,60]. Evidence from J. Yang et al. suggests that this limits the ability to create complex shapes like hollow structures and curved surfaces, despite their importance in applications like biomedical implants [54].

Another approach, while challenging for pure PTFE, is electrospinning, which creates fibrous structures [21]. Due to PTFE’s difficult processability, this often involves using a carrier polymer (like polyethylene oxide, which is later removed by sintering) or blending PTFE powder with other polymers like polyvinylidene fluoride (PVDF), as can be inferred from P. White et al.’s research. Electrospun PTFE/PVDF composite fibers have been investigated by P. White et al. for wearable TENGs, where the high electronegativity of PTFE is combined with the piezoelectric properties of PVDF [21]. Accordingly, as the study emphasizes, adding PTFE powder to the PVDF solution increases fiber surface roughness and can enhance TENG output [21]. Based on the above-mentioned studies, it can be concluded that the optimum percentage of PTFE loading in the PVDF matrix can significantly enhance the voltage and current output [21].

The advantages of using PTFE in these applications include its excellent dielectric properties, strong negative triboelectric polarity, low friction, high charge storage stability, chemical inertness, biocompatibility, and thermal stability [53,54,55,57,58,60,66]. Its position at the extreme negative end of the triboelectric series makes it an ideal candidate for creating large charge separation when paired with a tribopositive material, as shown by N. Wang et al. [52].

The study conducted by P. Johansson et al. and V. Singh et al. highlights PTFE’s limitations, including the inherent poor wear resistance of unfilled PTFE, which can be poor, especially at higher sliding velocities [56,67]. While fillers like carbon fibers or ceramics can improve wear resistance, they may increase the coefficient of friction or affect tribofilm formation [56,68]. PTFE’s difficulty in processing and bonding is a major challenge, particularly its low surface energy and natural liquid repellence, which impedes adhesive spreading and wetting [54,55,58,60,66,69]. Furthermore, while surface roughness can enhance triboelectric output, referring to the investigation by J.L. Armitage et al., it can also introduce mechanical problems caused by wear and abrasion over time [70].

#### 3.1.3. Ecoflex^®^

Ecoflex^®^ is an available dielectric silicone rubber manufactured by Smooth-On company (Macungie, PA, USA), which is usually used in biomechanical research and numerous flexible electronic applications because of its favorable biomedical properties [17,71,72]. These properties include non-toxicity, non-reactivity, and biocompatibility, which collectively reduce the risk of human tissue rejection and inflammation, making Ecoflex^®^ suitable for integration with the human body, as shown by Z. Liao et al. [71].

Ecoflex^®^ is identified as a silicone polymer, likely based on polydimethylsiloxane, and may contain silica particles to some extent, as shown by J. Lavazza et al. [17]. As reported in the literature, it is a two-part silicone elastomer catalyzed by platinum, which cures at room temperature and is commonly prepared by mixing the components in a 1:1 weight or volume ratio [17,72,73,74,75,76]. According to the findings of Z. Liao et al. and D. Rusu et al., the curing time is generally around four hours, although some variants like Shore 00-35 cure much faster [72,75].

A key characteristic of Ecoflex^®^ is its remarkable mechanical compliance, which closely matches that of biological tissues such as human skin, as shown by Z. Liao et al. [70,71]. This is provided by its high stretchability and durability, as shown by Z. Liao et al. [71]. Ecoflex^®^ materials come in various Shore hardnesses ranging from Shore 00-10 to Shore 00-50, representing a wide range of stiffnesses [72,75,76]. According to Z. Liao et al., Ecoflex^®^ exhibits very low pre-curing viscosity and can undergo extensive stretching without tearing [72]. Specific quantitative examples of mechanical properties shown in Z. Song et al.’s work, include a fracture elongation rate of up to 574% and a Young’s modulus as low as 0.2 MPa [77]. In K. Aw et al.’s work, Ecoflex^®^ 00-30, was found to show virtually no decrease in nominal stress when held under 200% strain for three hours, indicating good stability under prolonged strain, although stress relaxation over very prolonged periods or many thousands of cycles is a general consideration for elastomers [78]. Biocompatibility and softness also mean it shows no sign of irritation or allergic reaction upon long-term skin contact, unlike some traditional medical electrodes, as shown by A. Tessier et al. [79]. Table 3 summarizes AM-based fabrication strategies using Ecoflex^®^ in TENGs, highlighting surface engineering and output performance.

Polymers like Ecoflex^®^ play a significant role in TENGs due to their exceptional triboelectric properties [16]. Ecoflex^®^ serves as a flexible substrate or an active triboelectric layer in many TENG designs outlined by X. Wang et al., P. Pandey et al., Q. Zheng et al., S. Yang et al., A.L. Freire et al., J. Zou et al., K. Eltoukhy et al., and C. Jia et al. [3,11,74,80,81,82,83,84]. Its flexibility and stretchability are crucial for harvesting energy from mechanical motion in the surrounding environment, particularly in wearable or flexible devices, as shown by M. Shanbedi et al. and A.L. Freire et al. [16,82]. Ecoflex^®^-based TENGs have been developed for applications such as gait monitoring alarm systems by Q. Zheng et al. and self-powered sensors for tracking human motion information by C. Jia et al. [83,84].

While most sources do not explicitly detail the direct additive manufacturing of the active triboelectric Ecoflex^®^ layer itself, AM techniques, particularly 3D printing, are frequently used in conjunction with Ecoflex^®^ for fabricating TENGs and sensors. Three-dimensional printing is commonly used to create molds for casting Ecoflex^®^ samples or entire sensor structures (e.g., Z. Liao et al., D. Rusu et al., D. Ahmad et al., J. Zou et al., K.A. Eltoukhy et al., L. Migliorini et al., H. Huang et al., and E.R. Cholleti et al.) [72,75,76,80,81,85,86,87]. For example, in Z. Liao et al.’s study, the customized 3D-printed molds were used to produce uniaxial experimental specimens [71]. In their study, D. Rusu et al.’s negative frames for molding multiple specimens were also created using a 3D printer [75]. Molds with specific prismatic structures for hierarchical composite films were prepared by J. Zou et al. using 3D printing [80]. Closed 3D-printed molds were used by Eltoukhy et al. for injecting and curing Ecoflex^®^ layers in nanogenerator fabrication processes [81].

However, evidence from P. Fryń et al. and A. del Bosque et al. suggests that pristine Ecoflex^®^ is insulating [88,89]. To achieve conductivity or enhance triboelectric output according to S. Yang et al., A. Freire et al., C. Jia et al., L. Migliorini et al., T. Truong et al., P. Fryń et al., D.W. Kim, F. Wang, and B. Wicklein et al., it typically requires the incorporation of fillers or surface modifications [9,14,15,82,84,85,88,90,91]. Achieving a uniform distribution of fillers like carbon nanotubes (CNTs) in the Ecoflex^®^ matrix is important for consistent electrical conductivity and mechanical reinforcement, as shown by A. del Bosque et al. and M. Wang et al. [89,92]. Some specific Shore hardnesses may pose challenges in manual fabrication due to their rapid curing time, as shown by Z. Liao et al. [72]. While Ecoflex^®^ shows good stability under strain, the potential for stress relaxation in elastomers under very long-term or high-cycle use is a general consideration, though some studies like K. Aw et al. indicated minimal stress relaxation for specific Ecoflex^®^ types under moderate strain [78]. The manufacturing processes, while often low-cost and scalable (e.g., casting), may require specific techniques like vacuum degassing to the removal air bubbles, which can affect mechanical characteristics and porosity control [72,75,93,94].

#### 3.1.4. Polyamide Film (Kapton^®^)

A widely recognized polyimide film developed by DuPont, Kapton^®^ is a material frequently employed in advanced flexible electronic applications and sensors due to its desirable physical and chemical properties, as shown by N. Khomiakova et al. [95]. The standard Kapton^®^ film, specifically Kapton-H, is synthesized from pyromellitic dianhydride (PMDA) and 4,4′-oxydianiline (ODA) [96,97], corresponding to the molecular formula C_22_H_12_N_2_O_5_ [98]. It exists as a semi-crystalline polymer with a triclinic crystal structure [99] and is available in various thin sheet formats, with thicknesses ranging from approximately 50 µm (D. Jiang et al.) to 125 µm (S.K. Gupta et al.) or even thinner, such as 7.5 µm (S.K. Gupta et al.), 19.1 µm, and 12.7 µm thicknesses, as noted by Y. Fang et al. study in the case of Kapton^®^ HN films [96,99,100,101].

The essential characteristics making Kapton^®^ suitable for flexible and potentially biocompatible devices include its good mechanical durability and excellent mechanical properties, such as a tensile modulus of around 2.5 GPa, as indicated in S.K. Gupta et al.’s study, and a tensile strength of 230 MPa, as indicated by N. Khomiakova et al. [95,99,102]. It exhibits high thermal stability, with a glass transition temperature (Tg) between 360 °C and 410 °C (S.K. Gupta et al.) and an upper working temperature range of 250–320 °C, as shown by N. Khomiakova et al. [95,99]. Kapton^®^ also possesses good dielectric properties, with a dielectric constant of 3.4 and a low dissipation factor of 1 × 10^−2^ (M. Khelifa et al.) [100,101] and has shown a low outgassing in a vacuum [95]. Importantly for biomedical applications, the Kapton^®^ substrate is stated to be biocompatible (M.A. Signore et al.), making it a suitable material for wearable sensors intended for biomedical use [102]. As outlined in the studies conducted by M. Khelifa and E. Plis, chemical resistance and thermal stability are also highlighted benefits [100,103].

In the context of TENGs, Kapton^®^ is classified as a dielectric polymer and is commonly utilized as a triboelectric layer, as shown by M. Wang et al. [104]. It is positioned on the tribonegative side of the triboelectric series, making it prone to becoming negatively charged during contact electrification [11,105].

Regarding compatibility with additive manufacturing methods, polyimides, the material class of Kapton^®^, have been explored for 3D printing, as shown in C.B. Arrington et al.’s study [106]. Specifically, methods involving the 3D printing of polyimides followed by pyrolysis to create carbonaceous objects [106], and the 3D printing of all-aromatic polyimides have been reported in C.B. Arrington et al.’s study. While the direct 3D printing of Kapton^®^ film for surface texturing is not explicitly described, the fact that polyimides can be 3D-printed suggests potential routes for creating textured Kapton^®^ structures or composites using AM. Techniques like electrospinning, described by A. Bindhu et al., which produce nanofibrous, textured surfaces suitable for TENGs, have also been applied to polyimide-containing materials, such as polyamide, PI/PVDF composite nanofibers [11]. Ultraviolet, UV laser patterning on Kapton^®^ [103,106] can also be considered an AM-related surface modification technique. The use of Kapton^®^ as a flexible substrate for sputtered layers [102,107] aligns with common practices in flexible electronics fabrication that can be integrated with AM approaches.

For biomedical and wearable triboelectric systems, Kapton^®^ offers several advantages. Its established biocompatibility [102], excellent mechanical properties and durability as a flexible film [95,96,99,108], thermal stability [95,100,108], and chemical resistance [100,103] make it a robust substrate or component. Also, its surface can be modified through various techniques to tailor properties like printability [101] or integrate functional layers [102,109], which is essential for fabricating complex wearable devices. Even so, a significant limitation is the relatively low triboelectric output performance of standard Kapton^®^ film compared to some alternative or modified polymers [97]. As can be inferred by A. Bindhu et al.’s study, like many polymer-based TENGs, the output can be affected by environmental factors such as humidity [11]. Therefore, while Kapton^®^ is a promising material for flexible, biocompatible systems due to its inherent properties and processability, enhancing its triboelectric performance, likely through surface texturing, chemical modification, or composite formation, is necessary for its effective use in high-performance wearable TENGs. Table 4 summarizes the key fabrication strategies, surface modifications, and functional outputs reported for Kapton^®^-based TENG systems with relevant biomedical applications.

#### 3.1.5. Nylon

Characterized by the presence of polyethylene segments separated by peptide units, nylon polymers can establish strong intermolecular hydrogen bonds, contributing to their notable properties, including high resistance to temperature and chemicals (M. Arioli et al.), excellent toughness, flexibility, high modulus, strength, and abrasion resistance, as shown by D. Siddharth et al. [1,114,115,116,117,118,119,120,121,122,123].

Nylon-11 and Nylon-12 are used more often in applications like metal object coatings, tubing extrusion, or injection molding, as S. Dasgupta et al. mention [115]. Nylon-6,10 is described as a commercially important thermoplastic polyamide in M. Moniruzzaman et al.’s study [119], while Nylon 612 is noted for its high resistance to greases, oils, fuels, hydraulic fluids, water, alkalis, and saline, good stress cracking and abrasion resistance, low sliding friction coefficients, and high tensile and flexural strength, as shown by F. Calignano et al. [120]. The selection of a specific nylon type influences properties such as the melting point, low-temperature impact strength, moisture absorption, and chemical resistance [115].

Nylon has proved to be suitable for biomedical applications as reported by M. Arioli et al. and P. Le Bars et al. [1,122]. Nylon has also been involved in 3D bioprinting to develop scaffolds, joint implants, or drug carriers with tailored properties, as mentioned by M. Arioli [1]. Specifically, E. Kovrigina et al. developed Nylon-6-coated magnetic nanocomposites and nanocapsules with a Nylon-6 membrane, owing to the material’s well-documented high biocompatibility [124]. Therefore, in the context of TENGs, nylon is particularly relevant due to its tribopositive nature and functional performance [1,52], as shown in Table 5.

Because of this, nylon is an effective material for friction pairs when combined with tribonegative materials, such as fluoropolymers (e.g., polytetrafluoroethylene—PTFE) or polyimide, which are located at the bottom of the sequence and easily acquire negative charges [1,52]. Hence, N. Wang et al. identify that the greater the distance between two materials in the triboelectric sequence, the more charge they will generate upon contact [52]. Recent studies, like the ones conducted by M. Arioli, have focused on utilizing nylon surfaces as positive triboelectric materials [1]. For example, electrospun nylon fibers have been combined in M. Arioli et al.’s study with polyvinylidene fluoride (PVDF)/Molybdenum diselenide (MoSe_2_) fibers in a hybrid nanogenerator device, achieving notable open-circuit voltage and short-circuit current peaks [1].

The performance of nylon in triboelectric systems is influenced by its surface properties, which can be manipulated through various methods [114,118,126,127]. Nylon’s typically low surface energy and weak chemical reactivity can limit its adhesion with other substances, as shown by M. Rodríguez et al. [118]. Therefore, modifying surface properties like chemical composition, hydrophilicity, and roughness is often necessary for successful applications, including enhanced interfacial adhesion in composites or tailored interaction in triboelectric systems [114,118]. Surface modification techniques applied to nylon reported by F.J. Xu et al. include plasma treatment, UV irradiation, N-alkylation, potassium peroxydisulfate oxidation, and chemical grafting [127]. Plasma treatment is one example of treatment that can be applied to Nylon 6,6, recycled to enhance its surface properties according to M. Rodríguez et al. [118]. The chemical immobilization of nanoparticles onto nylon fibers has also been demonstrated by O.Y. Antonova et al., involving plasma pretreatment to oxidize the surface, followed by coating with a polymer and crosslinking [128]. Nylon 6 surface structure has been described by Q. Zhou et al.’s study, for example, revealing that crystalline morphology on the surfaces can differ from the bulk [126]. In P. Čapková et al.’s research, the phase composition and zeta potential of Nylon-6 nanofibers, influenced by manufacturing parameters, also impact their surface chemistry, which is relevant for subsequent chemical modification [129]. While most studies discuss the influence of surface modifications on general properties like adhesion or drug loading capacity [1,114,118,122,124,127,128,129,130,131,132,133], their direct impact on triboelectric charging characteristics is less detailed in the recent specialized literature, although the importance of surface charge distribution and stability in triboelectrification is generally acknowledged [133,134].

AM technologies offer promising avenues for producing nylon parts, including those for biomedical and triboelectric applications, allowing for the fabrication of components with complex geometries and tailored internal structures [81,132,135]. Common AM methods for nylon include Fused Filament Fabrication (FFF) and Selective Laser Sintering (SLS) [1,116,121,132,136]. Nylon-12, Nylon-6, Nylon 612, and nylon–carbon filaments are used in FFF [1,116,120,121,137], while PA12 powder is used in SLS [132]. This manufacturing technology of nylon allows for control over parameters such as infill density and pattern, layer thickness, and cooling rates, which significantly influence the resulting part’s microstructure, porosity, mechanical properties, and surface finish [120,121,132,137]. SLS involves processes like particle coalescence, solidification, and crystallization, influenced by laser and polymer powder properties according to G. Balan et al. [132]. FFF results in inherent layer-by-layer structures and potential anisotropy [120,132,136,137,138,139]. The surface quality and roughness of AM nylon parts are critical, as they impact friction, wear, and potentially triboelectric performance [122,132,137,139,140]. Research into controlling surface chemistry and charge distribution on AM nylon surfaces could further unlock its potential in wearable and implantable triboelectric systems [1,133,134].

#### 3.1.6. Polyethylene Terephthalate (PET)

Numerous studies have shown that PET possesses good mechanical strength, transparency, solvent resistance, and a large strength-to-weight ratio [141,142,143]. These properties mentioned above have led to widespread use across heavy industry and particularly in biomedical engineering applications such as artificial cell culture substrates, heart valves, sutures, and blood vessels, as shown by Y. Jiang et al. [141,143].

Despite its desirable bulk properties, K. Szafran et al. mentioned that the inherent surface characteristics of PET present challenges for certain applications, particularly in biomedical contexts and triboelectric energy harvesting [141,143]. Y. Jiang et al. and K. Szafran et al.’s studies outlined that PET surfaces have very few available functional groups, resulting in low surface free energy, poor wettability, and limited compatibility with biological environments [141,143]. This can lead to adverse biological reactions such as inflammation and thrombus formation, as shown by K. Szafran et al. [141,143].

Regarding TENGs, polyethylene terephthalate serves as a crucial component, as outlined in Table 6. According to M. Keykha et al., TENGs require two materials with substantially different electrical properties when brought into contact: one material induces a positive charge while the other induces a negative charge [144]. As a result, PET functions as an electron acceptor (or electron receiver) in a triboelectric pair [144]. PET offers advantages in TENG structures due to its significant stability and desirable flexibility, and its substrates can also be used as flexible supports in fiber-structured nanogenerators [145] and can be made suitable for electronic fibers by depositing a conductive film or polymer, as shown by V.N. Thankur et al.

Referring to the investigations by N. Lim et al. and S.H. Shin et al., the tribocharging behavior and overall output performance of PET-based TENGs are significantly influenced by the characteristics of their surface, including roughness, texture, porosity, and chemical composition [150,151,152]. Surface modification techniques are employed to tailor or enhance these properties, as shown in K.K. Yu et al.’s study [150,151,153].

Lately, the use of recycled PET in TENGs and other applications is gaining attention due to environmental awareness [142,148]. Therefore, as can be inferred from X. Li et al.’s investigations, recycled PET from sources like beverage bottles or waste clothes can be processed (cleaned, cut, ground) and used as the primary material [142,146]. Recycled PET can be electrospun into nanofibrous webs according to S. Yang et al.’s study [142] or used to produce PET aerogels (W.L. Lai et al.) for TENG fabrication [148]. Using recycled materials alleviates waste volume, which can present challenges, as processing steps like mechanical recycling can introduce contaminants and lead to macromolecular structure degradation, which influences material properties [148,154]. Despite these challenges, using recycled PET offers the significant benefit of reducing waste and boosting recycling rates [148,154].

M. Kujawa examined the PET-G that was 3D-printed and evaluated its surface topography subsequent to abrasion damage [155]. Although the study is not directly related to tribological properties, it can provide substantial information related to the surface properties of this material. In X. Zhang et al. and B. Reisinger et al.’s studies, texturing methods are mentioned for polymers (like hot-press molding for PP [156] or extreme ultraviolet (EUV)/laser for PET [157]) but are still not related to AM processes. Therefore, combined methods may be integrated in future studies to improve PET texture surfaces.

For biomedical, wearable, or implantable energy-harvesting applications, PET offers the advantage of being an established material in biomedical devices with good mechanical properties [141,142]. In studies like those conducted by V.N. Thankur and H. Ye, its use as a flexible substrate for electronic fibers and fabrics is also beneficial for wearable applications [145,158]. The potential to generate significant power through triboelectric mechanisms, especially with optimized surface texture and functionalization [151,152], makes it suitable for powering small-scale devices [148,149]. Surface modification techniques are vital not only for enhancing triboelectric performance but also for addressing PET’s poor intrinsic biocompatibility, wettability, and thrombogenicity [141,143]. Some modified PET materials have also demonstrated antibacterial properties, which are a valuable trait for biomedical applications, as shown by N. Stoyanova et al. [146,159]. Nevertheless, there are some limitations that include PET’s inherent hydrophobicity and poor biocompatibility requiring modification [141,143], potential performance degradation when using recycled material [148], and environmental considerations related to wastewater and energy consumption during processing [148].

#### 3.1.7. Comparative Assessment of AM Integration Potential for Synthetic Biocompatible Polymers

PDMS demonstrates the highest AM integration potential due to its established compatibility with various AM techniques such as DIW and DLP, which offer precise control over microarchitecture and high resolution. Its low processing cost, inherent flexibility, and adaptability with sacrificial templates/molds further enhance its versatility for creating complex TENG structures with tailored porosity and roughness. The material’s proven biocompatibility and long-term tribocharge stability are also critical advantages.

Ecoflex^®^ closely follows PDMS due to its remarkable mechanical compliance, high stretchability, and excellent biocompatibility. While its primary AM integration involves 3D-printed molds for casting, this method effectively enables complex and textured TENG designs. Despite its insulating nature requiring fillers, its demonstrated high triboelectric outputs (e.g., 30 V for porous vs. 23 V for nonporous) and robust durability validate its strong potential for wearable TENGs.

Kapton^®^ is a robust material with excellent mechanical, thermal, chemical stability and established biocompatibility. Even so, its direct AM for creating specific surface textures for biocompatible TENGs is not widely reported, it primarily functions as a flexible substrate for other patterning techniques (e.g., inkjet printing of functional layers). The significant limitation is its relatively low intrinsic triboelectric output performance (4–5 times less than engineered polyimides), implying that while is a reliable substrate, achieving high-performance TENGs requires substantial external surface engineering, making it less inherently adaptable for direct AM-driven triboelectric enhancement compared to the other materials.

Nylon ranks highly due to its strong compatibility with well-established AM techniques like FFF and SLS, enabling fabrication of components with complex geometries and tailored internal structures. Its inherent toughness, flexibility, and high biocompatibility make it suitable for biomedical applications. The ability to control nylon surface quality via AM and post-processing (e.g., laser polishing) suggests significant potential for optimization.

PTFE possesses exceptional triboelectric properties (Table 7), high charge storage stability, and chemical inertness, making it a strong candidate for TENGs. AM techniques (VPP/DLP, DIW, electrospinning) have been employed to fabricate complex microstructures and improve triboelectric output (e.g., voltage increase from ~85 V to ~320 V). Nevertheless, its high melt viscosity and poor flowability present significant challenges for AM processing, requiring the development of specialized inks and techniques. Its poor wear resistance and inherent hydrophobicity also necessitate further surface modifications for biomedical applications.

PET offers good mechanical properties and flexibility, with established use in biomedical devices. Its potential for sustainable AM fabrication from recycled materials and via electrospinning is a notable advantage. Surface modifications dramatically enhance their triboelectric output and improve biocompatibility. Despite its advantages, its inherent poor biocompatibility and wettability necessitate significant surface treatment, and AM texturing is not as broadly developed or detailed as with other materials. Challenges with recycled material degradation also exist.

### 3.2. Carbon-Based Biocompatible Materials

#### 3.2.1. Graphene

This monolayer structure is derived from graphite [162,163,164,165]. Graphene is a two-dimensional material with exceptional properties, including extremely high in-plane stiffness and superior (highest ever measured) strength [162,166]. According to K. Tadyszak et al., it has a Young’s modulus of approximately 1.0 TPa [164] and is nearly 200 times stronger than steel (S.R. Laraba et al.), while at the same time being more elastic than rubber [166]. Among carbon-based nanomaterials, graphene shows outstanding electron transport properties, with electron mobility nearly 100 times faster than silicon and an intrinsic mobility of 200,000 cm^2^·v^−1^·s^−1^ (K. Tadyszak et al.) [162,164,166]. Graphene demonstrates outstanding thermal conductivity (~5000 Wm^−1^·K^−1^ [164], two times that of diamond), a large theoretical specific surface area (2630 m^2^·g^−1^, according to K. Tadyszak), high optical transmittance (~97.7% [164]), and is hermetic, preventing even the smallest atom from passing through a defect-free single layer [164,165,166]. Because of its pristine graphene, common derivatives include graphene oxide and reduced graphene oxide [166,167], which are more prevalently researched for biomedical applications due to their easier manipulation and ability to form stable aqueous suspensions compared to pristine graphene’s hydrophobicity, as shown by S. Syama and P.V. Mohanan [167].Stemming from its multifunctional physicochemical and biological properties, including mechanical strength, electrical conductivity, thermal conductivity, large surface area, and chemical stability [163,164,165,166], graphene and its derivatives have garnered significant attention for a broad range of applications, particularly in biomedical fields [163,164,166,167,168,169]. Graphene’s biocompatibility is a crucial consideration for biomedical applications, especially when used as a scaffold material, as shown in the study by L.A. Lazăr et al. [165]. Studies have shown potential for applications like drug delivery and tissue engineering [164,166,167,168], while cytotoxicity remains a significant concern that could limit their use according to S.R. Laraba and S. Syama’s studies [166,167]. The toxicity of graphene-based materials is reported by K. Tadyszak to be strongly dependent on factors such as flake size, structure, shape, surface chemistry, and the specific cell type being assessed [164,165]. Functionalization methods are considered necessary to modify graphene’s chemical reactions (A.I. Lazăr et al.), ideally through environmentally friendly and bioinspired methods, to potentially eliminate toxicity and side effects [165,166]. Consequently, inconsistent results regarding cytotoxicity in various studies highlight the need for further research to fully understand the correlation between these factors and potential adverse biological effects [165]. Table 8 summarizes engineering strategies and applications of graphene materials in TENGs.

Evidence from B. Xie et al. and S. Kim et al.’s studies suggests that graphene is well-suited for use in TENGs [173,174], functioning as either an active triboelectric material or a transparent and flexible electrode [174]. R. Zhang et al. emphasize in their study that its utility in TENGs stems from its notable characteristics, including high electron mobility, large surface area [175], flexibility, and the ability to store electric charges [174]. Reviews such as S.M. Kim, A.N. Parvez et al., D. Deepak, and S.S. Roy specifically address the applications of graphene in TENGs [173,176,177]. Graphene has been incorporated into various TENG configurations, including conductor-to-dielectric and metal-free dielectric-to-dielectric geometries by M.G. Stanford et al., and single-electrode composite designs [178]. For example, R. Zhang highlights that aligning graphene sheets within a PDMS matrix has been shown to improve the output performance of TENGs [175,179,180]. Composites like PVDF–graphene nanofibers have been prepared for TENGs by Y. Jiao et al., demonstrating enhanced performance with optimized graphene content [172].

3D printing provides versatile approaches for fabricating graphene-based structures with controlled surface characteristics for triboelectric and biomedical applications. Laser-induced graphene (LIG) is highlighted by M.G. Stanford as an AM technique where infrared laser irradiation converts polymer surfaces, such as polyimide or cork, into porous graphene [178]. Therefore, LIG composites function as triboelectric materials and can be used to fabricate TENGs, including flexible and stretchable single-electrode devices by embedding LIG in PDMS [178]. Following the M.G. Stanford study, it reveals that this technique allows for the creation of LIG on diverse substrates like PI, cork, and even wood [178], demonstrating its adaptability for various material platforms. Beyond LIG, 3D printing and bioprinting are explored for creating graphene-based structures and scaffolds, particularly relevant for biomedical platforms like tissue engineering [164,181,182]. Three-dimensional porous macrostructures can also be fabricated from graphene-based aerogels via self-assembly, as shown by E. García-Tuñón et al. [183]. Other processes relevant to AM, such as extrusion via a twin-screw extruder, have been used by A. Poosala et al. to prepare graphene nanocomposites [184], and electrospinning has been employed to create PVDF–graphene nanofibers [172]. Direct ink writing is also mentioned in E. García-Tuñón et al.’s study as a method for creating 3D ceramic structures [183], a technique applicable to graphene-based inks.

For biomedical, wearable, and implantable triboelectric applications, graphene-based TENGs offer several important advantages. Their inherent flexibility and potential for transparency make them suitable for wearable sensors and electronics [165,166,172,174,178]. Graphene’s excellent mechanical and electrical properties contribute to the robustness and performance of these devices [164,166,168], and they can potentially serve as continuous power sources for self-powered sensors [172]. Albeit the primary limitation remains that the potential cytotoxicity and toxicity concerns associated with graphene materials highlighted in S.R. Laraba and S. Syama’s studies [166,167] are influenced by material properties such as flake size and surface chemistry [164,165]. Successfully mitigating these toxicity issues, likely through functionalization and careful material design [165,167], is critical for enabling widespread safe use in implantable or long-term wearable biomedical devices.

#### 3.2.2. Carbon Nanotubes (CNTs)

Carbon nanotubes, generally known as CNTs, are a notable allotrope of carbon, garnering significant attention in nanotechnology due to their exceptional properties [185,186]. Their structure may be envisioned as a graphene rolled-up sheet (S. Yazdani et al.) of a planar hexagonal lattice of carbon atoms [186,187]. According to N. Gupta et al., CNTs are typically sp^2^ hybridized and possess a very high aspect ratio, with diameters in the nanometer range and lengths extending to micrometers, potentially yielding a length-to-diameter ratio exceeding 1,000,000 [185,186,188,189]. This unique geometry, coupled with their inherent properties, makes them highly relevant for developing advanced materials, including those for triboelectric applications.

CNTs are usually classified into two main types based on the number of concentric graphene layers: single-wall carbon nanotubes (SWCNTs) and multi-wall carbon nanotubes (MWCNTs) [187]. According to the literature, single-walled carbon nanotubes (SWCNTs) are composed of a single rolled graphene sheet, typically exhibiting diameters between 0.4 and 3 nm (S. Yazdani et al.). As the study reveals, MWCNTs, on the other hand, are composed of several concentric graphene layers, resulting in larger diameters typically between 1 and 200 nm [187].

From an electrical point of view, CNTs can manifest both semiconducting and metallic behaviors, encompassing the full spectrum of properties important for technological applications, as shown by R. Hirlekar et al. [190]. These advanced materials are known for their excellent electrical conductivity, reported in S. Ghoshal’s review to range from 10^2^ S/m to 10^7^ S/m at 300 K [185,188,189,191]. This high conductivity may represent an advantage for charging dissipation and accumulation control in general triboelectric devices. From a mechanical point of view, CNTs possess good tensile strength [185], and K.A. Wepasnick reported them to have exceptionally high values, potentially up to 100 times greater than steel [189]. Quantitatively, according to S. Ghoshal, they exhibit a high tensile modulus (270 GPa–950 GPa [191]) and tensile strength (11 GPa–63 GPa [191]), with a modulus of about 1 TPa indicated in C.O. Ujah et al.’s review [192]. These mechanical properties make them promising for reinforcing materials and creating flexible or structurally robust components [188,192,193]. CNTs also have extremely high surface areas [188,189], which is a crucial parameter influencing interactions and charge exchange at material interfaces, particularly relevant for triboelectric effects.

The use of CNTs in biomedical, flexible, or implantable devices necessitates the careful consideration of their biocompatibility. As mentioned by R. Hirlerak et al., pristine (non-purified or non-functionalized) CNTs often contain impurities such as amorphous carbon and metallic catalyst nanoparticles (e.g., Iron, Cobalt, Nickel, and Molybdenum), which can pose severe toxic effects [190]. The intrinsic toxicity is influenced by the degree of surface functionalization and the nature of functional groups because structural factors like fiber shape, length, and aggregation status can affect their deposition and subsequent biological responses in the body [190]. S. Yazdani outlines that MWCNTs tend to accumulate more in living organisms compared to SWCNTs, making SWCNTs generally preferred for pharmaceutical applications [187]. According to M. Văduva, issues related to dispersion and agglomeration in physiological environments can negatively impact the biocompatibility of CNTs [194]. A common strategy to enhance their solubility and improve biocompatibility is functionalizing CNTS covalently or non-covalently [187,189]. The functionalization process can introduce groups like carboxylic acid or amine groups, making them more suitable for biomedical applications such as drug and gene delivery [187,193]. Despite these efforts, the presence of CNTs in medical applications remains a complex and somewhat controversial topic, requiring further interdisciplinary research [188]. Some studies mentioned by A. Saberi suggest that immobilized CNTs within a metallic biomaterial matrix can prevent their direct deposition into surrounding tissue, potentially enhancing biosafety [188]. CNTs have also been investigated for nerve injury treatment, attributed to their negative charge and ability to facilitate nerve cell reconnection, as shown by A. Saberi [188].

CNTs have shown promise in triboelectric systems, with studies showing their use in triboelectric nanogenerators and other triboelectric applications, as highlighted in Table 9 [195,196]. For example, in J. González et al.’s study, CNTs serve as nanocarbon fillers in cellulose–nanocarbon composite films designed for TENG and PENG (piezoelectric nanogenerator) devices [196]. Studies like those conducted by M.V. Il’ina et al. have demonstrated triboelectricity generation from vertically aligned carbon nanotube arrays and their application in flexible triboelectric nanogenerators for self-powered weighing systems [195]. The performance of these triboelectric devices is not solely dependent on the filler’s electrical conductivity but is also influenced by factors such as the morphology, size, dimensionality, specific surface area, nanoscale dispersion, filler content, and matrix porosity of the composite components, as highlighted by González et al. [196]. This outlines the critical interplay between the CNT structure, dispersion, and the resulting surface characteristics in determining triboelectric output.

Additive manufacturing techniques provide versatile pathways for fabricating CNT-based materials and structures relevant to triboelectric applications. In A. Mora et al.’s research, Fused Filament Fabrication has been used to create CNT/polymer composites, including those based on PLA and high-density polyethylene (HDPE) [197]. Filament feedstocks in this study containing controlled CNT loadings are synthesized, often through melt blending, for 3D printing [197]. The electrical conductivity of these 3D-printed composites presented in A. Mora’s research increases with CNT loading, achieving low percolation thresholds (e.g., 0.23 vol% for CNT/PLA and 0.18 vol% for CNT/HDPE, as reported in the study [197]). Fused Deposition Modeling has been employed to fabricate nanocomposite filaments using CNTs and ABS polymer [198]. Printed ABS/CNT samples by B. Podsiadły et al. demonstrated improved tensile strength (by 12.6% at a high CNT content) and exhibited non-linear electrical properties attributable to CNT’s semiconductor nature and tunneling effects [198]. Resistivity values were reported as significantly decreased with an increasing CNT content (2.5 Ωm for 4.76 wt% CNT and 0.15 Ωm for 9.09 wt% CNT at constant voltage [198]). These CNT-based ABS composites developed by B. Podsiadły et al. have been considered promising for the FDM fabrication of structural elements, electronics, and sensors [198]. A particular challenge in the AM of CNT polymer nanocomposites, particularly methods like FDM, is the agglomeration of CNTs, which can lead to issues such as nozzle blockages and flux instability [191]. Accordingly, S. Ghoshal emphasizes that improved dispersion and interfacial compatibility between carbon nanotubes and the polymer matrix are crucial for effectively transferring the remarkable intrinsic properties of individual CNTs to the bulk performance of the printed composite [191]. Other printing-based methods outlined in T. Murakami et al.’s research, such as using organic CNT/paraffin ink, have been developed for fabricating microelectrode arrays, demonstrating the potential for patterned CNT structures via AM [199].

High electrical conductivity and mechanical strength are advantageous for device performance and durability [185,188,189,191,192] because their high aspect ratio facilitates conductive network formation at lower filler concentrations [197]. The ability to functionalize surfaces shown in the studies above allows for tailored interactions, improved dispersion, and potential integration with biological systems [187,193,200]. When properly incorporated, the studies show that they can enhance the properties of polymer or metal composites fabricated with AM [188,192,197,198]. Their role in influencing surface area, dispersion, and morphology is critical for optimizing triboelectric charge generation and transfer, as shown by González et al. [196].

**Table 9:** Engineering methods and application potential of CNT material in TENGs.

Fabrication Strategy	Surface Engineering	Functional Performance	Biomedical Applications	Reference
hemical Vapor Deposition (CVD) growth of vertically aligned CNT arrays	Aligned CNTs structured for triboelectricity.	Output: 3.2 V, 0.21 µA, 672 nW.	Self-powered weighing systems.	[195]
Blending CNTs with cellulose (microcrystalline cellulose–MCC, cellulose powder–CP, cellulose nanofibers–CNF); oven-dried films	Homogeneous nanocarbon dispersion, enhanced with MCC.	Max voltage: 39 V (MCC); current: 3 mA/m^2^; power: 60 mW/m^2^ at 40 MΩ.	Biodegradable nanogenerators for low-frequency energy harvesting.	[196]
Electrospun polyaniline (PANI)/CNTs/AgNWs composite electrode	Friction layer with hydrophobic surface (contact angle > 120°).	Voc ≈ 150 V; Isc ≈ 60 µA.	Liquid-type sensing systems.	[201]

Most studies have outlined that agglomeration remains a significant challenge during composite processing and in biological environments, impacting both performance and biocompatibility [187,191,192,193,194]. Also, the potential toxicity associated with pristine CNTs and catalyst residues necessitates rigorous purification and functionalization strategies to ensure safety in biomedical applications [188,190]. While functionalization highlighted by A. Saberi et al. shows that biocompatibility can be improved, the overall safety profile of CNTs for long-term implantation is still debated [188]. Achieving precise control over CNT dispersion, alignment, and integration within AM processes is crucial but can be technically demanding [191].

#### 3.2.3. Reduced Graphene Oxide (rGO)

Reduced graphene oxide or rGOs represent an intermediate structural form between graphene and graphene oxide (GO), achieved by partially restoring graphitic characteristics through the removal of oxygen-containing functional groups from GO [202]. This process, as described by R. Patil et al., aims to recover properties lost during the oxidation of graphite, specifically restoring the sp^2^ carbon structure [202]. When approaching the structure of graphene, rGO retains some residual sp^3^ bonds stemming from remaining oxygen functionalities [202,203,204,205]. The carbon-to-oxygen (C:O) ratio in rGO is typically higher than that of GO, indicating a reduced amount of oxygen-containing groups as outlined in R. Patil et al. and D. Perumal et al.’s studies [202,206]. Common functional groups present in GO, such as alkoxy (C-O-C), hydroxyl (-OH), carboxylic acid (-COOH), and carbonyl (C=O) groups (S. Ml. Majhi et al.), are partially eliminated during the reduction to rGO as mentioned in the studies above [202,205,206].

The reduction process of GO to obtain rGO can be achieved through various methods, including chemical, thermal, or electrochemical reduction techniques, often employing reducing agents such as sodium borohydride, hydrazine, or hydrogen gas [202,205,207,208]. Graphene oxide is commonly derived from graphite through synthesis methods such as Hummer’s technique (P. Viprya et al., P. Jagódka et al.) or Brodie’s methods (S.M. Majhi et al.) [205,208,209,210,211,212]. The selection of the reduction method and parameters from the specialized literature mentioned above significantly influences the quality and resulting properties of rGO [202].

As highlighted by R. Adami and D. Scarpa in their study, an important property of rGO relevant to its application in conductive composites and charge-related phenomena is its enhanced electrical conductivity compared to GO [203,213]. A. Mondal explains that this is due to the partial restoration of the sp^2^-bonded carbon network, enabling effective electron transport through the π* orbitals [214]. rGO possesses high electron mobility and conductivity, like graphene, although the presence of residual defects and oxidized chemical groups can modulate these electronic properties [203,214]. C. Wang et al. mention a specific electrical conductivity range of 600–900 S/cm for GO [215]; this value is typically associated with reduced forms or may refer to specific preparation methods, as GO generally exhibits high electrical resistance due to the insulating nature of its oxygen groups [215]. The literature findings support the conclusion that rGO is generally acknowledged to have better conductivity than GO [203,213,216]. The incorporation of rGO into matrices has been shown by A. Smirnov to enhance conductivity, with conductivity increasing as rGO content increases in composites [212].

Morphologically, rGO can present as crumpled thin sheets with a rougher surface and a wave-shaped corrugated structure [206,214]. It possesses a high surface area [213,214,217,218], with values up to 1780 m^2^/g reported by S.K. Samaniego Andrade for rGO-doped porous carbon structures [217]. An analysis of the literature reveals that the porosity and specific surface area of rGO are considered crucial factors for various applications [207,209,217,218].

The biocompatibility of rGO is an important consideration for its use in biomedical applications, including additively manufactured biocompatible materials. rGO has been reported by R. Patil to be biocompatible with various cell types, including human mesenchymal stem cells (hMSCs), making it a promising material for tissue engineering applications [202]. It has been proven by P. Wilczek et al. to be beneficial for cell growth [219] and has been explored in contexts such as tissue regeneration [202,220], nerve regeneration according to Z. Wang et al. [220], and as a potential material for dental and orthopedic bone substitutes, as highlighted by M.S. Kang [221]. Several studies, including that of D. Perumal, indicate that rGO exhibits lower toxicity compared to GO [206]. However, the cytotoxicity of graphene-based materials, including rGO, can vary depending on factors such as size, shape, concentration, aggregation, and, of course, surface properties [219]. Higher concentrations have been reported by P. Wilczek to cause cell apoptosis and lung granuloma in mice [219]. Small-sized GO was found in the same study to have greater hemolytic activity than aggregated graphene sheets, while compacted graphene sheets were more damaging to mammalian fibroblasts than less densely packed GO [219]. Hence, the careful consideration of rGO’s form, concentration, and surface characteristics is necessary when designing biocompatible materials.

When examining triboelectric behavior, rGO has shown promise, particularly in triboelectric nanogenerators. rGO has been observed by Y. Zhou to act as an electron-trapping site within the friction layer of triboelectric materials, leading to enhanced triboelectric effects [5]. The material’s structure, containing both oxygen-rich (more insulating) and oxygen-poor (more conductive/graphitic) regions, is beneficial for charge carrier accumulation, as shown by D. Scarpa et al. [213]. This leads us to the understanding that the presence of defects and oxidized chemical groups in rGO is thought to contribute to a higher electrical permittivity than GO (D. Scarpa et al.), which is crucial for facilitating the formation and stability of the electrical double layer, thereby enhancing capacitive performance [213]. Although primarily discussed for supercapacitors in D. Scarpa’s paper [213], these properties are also fundamentally important for understanding charge separation and accumulation in triboelectric systems. Beyond energy harvesting, rGO/GO nanosheets have also been investigated in the field of tribology as additives in lubricants, where they can reduce wear via deposition and film formation (M. Sarno et al.) and reduce friction through shear and lamination on contacting surfaces [222]. Based on the literature reviewed in this study, this demonstrates the inherent tribological activity of rGO sheets.

The incorporation of rGO into AM processes is being explored to develop functional composite materials, including those intended for biocompatible applications. M. Sieradzka et al.’s research proved that rGO can be added to polymer matrices like High-Impact Polystyrene (HIPS) [223] and PLA (M. Hanif et al. [224] and M.V. Silva et al. [225]) to create composite filaments for 3D printing [223,224,225]. GO gel inks have also been used by C. Ramírez in DIW to fabricate porous 3D structures, which are then thermally reduced to form structured rGO electrodes [226]. R. Patil and Z. Wang have incorporated rGO into 3D-printed scaffolds for applications such as nerve regeneration [202,220]. The concentration of rGO in the composite is important, for example, in M.V. Silva et al.’s study, 10% wt rGO in PLA resulted in poor electrical conductivity in 3D-printed electrodes unless surface treatments were applied to expose the conductive material [225]. Conversely, according to M. Sieradzka, a lower concentration (0.5 wt%) of rGO in HIPS improved the tensile strength and printed well without nozzle clogging [223]. Table 10 summarizes recent developments in rGO surface engineering strategies, performance metrics, and potential applications.

Using rGO in AM biocompatible systems for triboelectric applications offers several potential advantages. Its enhanced conductivity can impart electrophysiological properties to scaffolds [220] and improve the electrical performance of composite materials [212,223,224,225]. The inherent tribological activity and charge-trapping capabilities of rGO [5,214,222,227] suggest that rGO-loaded AM structures could function as or enhance the performance of TENGs for self-powered devices in biomedical contexts. The ability to create complex, porous 3D architectures using AM [226,228,229,230] allows for the design of structures with tailored surface areas and textures that could optimize triboelectric performance and integration with biological tissues. The rGOs also offer improved mechanical strength compared to GO [202] and can enhance the mechanical properties of composites [223,231].

Despite these advantages, certain challenges persist; achieving a stable and robust 3D architecture with rGO sheets can be difficult due to intrinsic internal mechanical fragilities, as shown by D. Scarpa et al. [213]. Maintaining a uniform dispersion of rGO within the polymer matrix during composite preparation and AM processing is crucial, as agglomeration can hinder performance [212,222,225]. Restacking rGO sheets remains a challenge in 3D graphene structures according to R. Singh [207]. The specific surface properties of different rGO preparations can vary, as shown in M. Sarno’s paper, meaning that each rGO/base material combination might require dedicated effort for optimization [222]. Beyond that, integrating rGO into composites via methods like DIW requires optimizing ink rheology and composition [226], while filament-based printing needs materials that avoid nozzle clogging [223]. Ensuring that the AM process does not negatively impact the critical properties of rGO, such as its conductivity and surface chemistry, is also important [225]. Despite these challenges outlined by the literature, the unique combination of tunable properties, conductivity, and demonstrated triboelectric relevance makes rGO a promising component for developing additively manufactured biocompatible materials with integrated triboelectric capabilities.

#### 3.2.4. Carbon Black (CB)

CB is a widely produced nanomaterial consisting of finely distributed particles of elemental carbon [232,233,234,235]. C.M. Long describes it as a product that is manufactured with controlled properties, distinct from combustion byproducts like black carbon [233]. According to G.A. Kelesidis, CB particles are generated through surface reactions and subsequent agglomeration during synthesis, resulting in clusters of primary particles or spherules [232]. These primary particles (globules) typically have diameters ranging from approximately 15 to 300 nm [234], forming strong, chemically bonded aggregates with equivalent diameters between 85 and 500 nm, as shown by V.A. Drozdov et al. [234,236]. As outlined by V.A. Drozdova et al., the aggregates described in their work, in turn, are linked by weaker Van der Waals forces into larger agglomerates, which can range from approximately 1 to 100 µm in size [234]. This structure of coalesced particles forming aggregates, highlighted also by C.M. Long, is known as aciniform or grape-like, and the aggregate is considered the smallest dispersible unit in CB products [233]. CB is characterized by S. So et al. study by its paracrystalline carbon structure and a high surface-area-to-volume ratio [235]. The specific surface area of different CB grades can vary significantly, ranging from approximately 6 to 1400 m^2^/g (V.A. Drozdov et al.), which exhibits an inverse correlation with the dispersity of the primary particles [234].

While some literature sources suggest that CB is known as an unsuitable biomaterial (S. So et al.), others highlight its potential use in biocompatible contexts [235]. For example, flexible and conductive composites have been prepared by N.P. Kim using biocompatible thermoplastic polyurethane (TPU) and carbon fillers, including CB, with suggestions that carbon fillers can improve electrical conductivity without losing biocompatibility in this matrix [237]. Nonetheless, it is worth emphasizing that CB was nominated for potential review in the Report on Carcinogens by the US National Toxicology Program in the 2012 edition [233], indicating potential hazard concerns that require careful scientific distinction from other carbon materials, as mentioned in C.M. Long et al.’s work [233]. The biocompatibility and tissue interaction of other carbon forms, such as carbon fibers, have been studied by R. Petersen, demonstrating potential advantages in biomedical applications like stimulating osseointegration [238], but this behavior is distinct from CB.

Within the framework of triboelectric nanogenerators and associated triboelectric systems, CB plays a significant role, primarily due to its conductive properties and influence on charge behavior. CB can be incorporated into polymer matrices to create conductive composites used in TENGs in studies conducted by A.L. Freire and P.K. Szewczyk [82,239]. The study of P.K. Szewczyk et al. demonstrates that the incorporation of CB yields varying effects depending on the polymer matrix: it reduced the triboelectric output of polyurethane and polystyrene by more than 90%, whereas it enhanced the output of polycarbonate by 260% [239]. This differential effect, according to the study mentioned above, underscores the importance of selecting the CB content carefully for optimal performance [239].

Consequently, as shown by P.K. Szewczyk, CB particles can act as charge-trapping sites, which increases the dielectric constant of the material [239]. This leads to an increase in the total amount of negative electrical charges in the TENG, consequently enhancing the power output [239]. In such multilayered TENGs, a CB-doped layer can function as a charge transfer or charge storage layer, significantly increasing the charge quantity and power output compared to devices without CB particles [239]. For example, the integration of CB by P.K. Szewczyk into an all-electrospun TENG composed of cellulose acetate (CA), polyether sulfone (PES), and polystyrene (PS) led to an increase in power output exceeding 250% [239]. The progressive incorporation by A.L. Freire of CB into bacterial cellulose films for metal-free electrodes in TENG prototypes shows a carbon black concentration-dependent electrical response, where reaching a critical filler concentration allows conductive grains to create pathways along the material, facilitating charge transfer and improving TENG performance, as shown by Freire et al. [82].

Based on the reviewed literature, the electrical conductivity in CB-filled polymer composites is largely determined by the percolation threshold, where enough conductive filler forms a continuous pathway through the material [196,236,237,240,241]. The formation of interconnected CB-based structures near or above the percolation threshold forms conductive networks that enhance electrical conductivity, as shown by V. Brunella et al. [241]. Consequently, the structure or aggregates and particle size of CB are principal properties influencing composite behavior [242,243].

The use of carbon black within additive manufacturing technologies is already well-documented and extensively applied, particularly for creating conductive polymer composites [235,237,244,245,246,247]. A. Koterwa and A.V. Papavasileiou highlight that CB/PLA is a common conductive composite used for 3D printing electrodes for electrochemical applications [236,248]. CB functions as a conductive filler within a range of polymer matrices, including PA12, as reported by N. Vidakis et al., TPU (N.P. Kim), PDMS (S. So et al.), and PC (V. Brunella et al.) [235,237,241,245]. These types of composites are processed using methods like melt mixing and extrusion to produce filaments for FFF 3D printing [237,245,247] or incorporated into slurries for material extrusion [235]. Applications of these 3D-printed conductive composites include electronic sensors, circuits, and electrodes [245,246,247]. Table 11 highlights the use of carbon black in TENGs, including fabrication methods, performance results, and biomedical applications.

#### 3.2.5. Comparative Assessment of AM Integration Potential for Carbon-Based Biocompatible Materials

Graphene offers exceptional TENG performance, with high output figures and diverse AM integration methods, especially the facile, solvent-free LIG technique. Its inherent flexibility and mechanical strength are also highly beneficial. Nonetheless, the persistent and often inconsistent cytotoxicity concerns, requiring significant functionalization and further research, somewhat limit its immediate widespread application in implantable or long-term wearable biomedical devices compared to rGO.

While possessing excellent electrical and mechanical properties that are valuable for TENGs and reinforcement, CNTs face substantial challenges in AM integration due to severe agglomeration issues, leading to difficulties in achieving uniform dispersion and print stability. Furthermore, the intrinsic toxicity of pristine CNTs and the ongoing debate regarding their long-term safety for biomedical applications place them lower on the ranking for biocompatible AM-enabled TENGs, despite functionalization efforts.

Reduced graphene oxide exhibits the most balanced profile for AM-enabled biocompatible TENGs. Its strong AM compatibility, particularly for DIW to create complex porous 3D structures, combined with its established role as electron-trapping sites leading to significant TENG performance enhancements, is highly advantageous. Critically, rGO is generally reported as biocompatible and less toxic than GO, making it highly suitable for biomedical integration. While dispersion and restacking remain challenges, the potential for tailored surface chemistry and high surface area makes it a top choice.

Carbon black demonstrates high AM compatibility (Table 12), being well-studied and cost-effective for creating conductive polymer composites via FFF and material extrusion. It can significantly enhance TENG output by acting as charge trapping sites and increasing dielectric constant. Nevertheless, its significant biocompatibility concerns, including its nomination for carcinogen review and designations as an unsuitable biomaterial by some sources, pose a considerable limitation for direct biomedical applications unless rigorously encapsulated. Dispersion and optimal concentration also remain key challenges for performance.

In summary, while all carbon-based materials offer unique advantages for TENGs, their suitability for AM-enabled biocompatible applications is largely dictated by their processability, ability to form controlled microstructures, and, most importantly, their safety profile when interacting with biological systems. rGO, with its superior biocompatibility and strong AM-TENG performance, currently leads this group within the scope of the provided study.

### 3.3. Advanced Biocompatible Materials and Enhancements

#### 3.3.1. MXenes (Ti_3_C_2_T_x_)

MXenes are described by A. Dey and J. Huang as a material derived from layered ternary MAX phases (M_n+1_AX_n_) through the selective etching of the A-group element layer [252,253,254]. Ti_3_C_2_T_x_ originates from the Ti_3_AlC_2_ MAX phase precursor and is the earliest discovered and most extensively investigated member of the MXene family [252,253,254].

As mentioned by A. Dey et al., J. Huang et al., and M. Malaki et al., the general structural formula for MXenes is M_n+1_X_n_T_x_, where M is an early d-block transition metal, X is carbon and/or nitrogen, and T_x_ denotes surface termination groups [252,254,255]. Therefore, based on findings in the research literature, these surface terminations are critical as they significantly influence the chemical reactivity, electronic properties, and interactions of MXenes with other substances [255,256,257,258]. For example, M.B. Bilibana outlined that the presence and ratio of these groups affect the material’s conductivity [256].

In recent years, MXenes have gained recognition as promising candidates for triboelectric nanogenerators, contributing to improved functionality and performance [259]. Z. Zhao et al.’s study highlighted their integration into both triboelectrification and electrode layers, leveraging their exceptional properties to improve electronegativity, charge density, electrical output, and introduce self-healing and stretchability [259]. The elevated electrical conductivity (e.g., 24,000 S·cm^−1^ for Ti_3_C_2_T_x_, as reported by M. Malaki and R.S. Varma) along with its surface chemistry enables efficient electron transport, which is essential for optimal TENG performance [255,259]. The layered structure and the nature of the surface terminations directly impact the charge density variations at the surface, which is fundamental to the tribocharging process [259]. Surface functionalization shown in W. Huang et al.’s work can be used to tailor electronic properties, including charge distribution and work functions [258].

The unique properties of MXenes, including their stability in water-based colloidal solutions with high surface charge (exhibiting a strongly negative zeta potential, typically exceeding −30 mV in magnitude, as shown by U.U. Rahman et al.), make them suitable for ink formulations and processing techniques like AM [257,260]. As shown by M.L. Matias, 3D printing enables the fabrication of high-performance devices with precise architectural control, providing opportunities to customize surface textures that influence triboelectric performance [260,261]. Additive-free 2D Ti_3_C_2_T_x_ ink has been successfully used as reported by S. Jung et al. and Y. Li et al. for 3D printing (e.g., micro-supercapacitors), demonstrating the feasibility of integrating this material into AM for creating complex structures [262,263]. Such a capability underpins the development of AM-fabricated biocompatible devices where controlled surface topography can enhance triboelectric effects. The triboelectric performance and biomedical applicability of MXene-based systems are summarized in Table 13, highlighting their fabrication strategies, functional enhancements, and use in energy-harvesting and wearable sensing technologies.

Among the advantages of employing Ti_3_C_2_T_x_ in this domain are its large surface area, excellent electrical conductivity, hydrophilicity, tunable surface chemistry, and mechanical properties like flexibility and stretchability [252,255,256,259,261,265]. Although MXenes have demonstrated promise in biomedical applications [252,254,256,257,260,261], certain challenges persist, particularly regarding restacking, oxidation, and large contact resistance. Oxidation susceptibility is a notable limitation [252,266,267], though strategies involving surface modification or doping can enhance stability and overall performance, including flexible and potentially implantable applications [252,256,262,268]. Tuning the cytotoxicity of Ti_3_C_2_ flakes through surface modifications has also been demonstrated in A. Rozmysłowska-Wojciechowska et al.’s work [269].

#### 3.3.2. Ionic Liquids and Hydrogels

Ionic liquid-based hydrogels, often referred to as ionogels, represent a significant advancement in developing soft ionic materials suitable for applications like TENGs [270]. Unlike conventional hydrogels, as mentioned by K. Sheikh and X. Qu, which use water as the solvent, ionogels incorporate ionic liquids (ILs) within a polymer network [270,271]. Ionic liquids are salts that exist in a liquid state at or near room temperature, composed entirely of ions, typically organic quaternary cations and inorganic or organic anions [270,272]. Therefore, according to the literature, this intrinsic ionic nature endows ILs with remarkable conductivity, thermal stability, and non-volatile characteristics [270,271].

Integrating ILs into hydrogel matrices combines the liquid properties of ILs with the structural integrity and flexibility of polymers [270]. The resulting ionic gels presented in K. Sheikh et al.’s work are described as stable, soft, and elastic materials [270]. They offer high ionic conductivity and low saturated vapor pressure, contributing to highly sensitive perception over a wide temperature range and a distinct thermal-sensitive feature for multimodal signal sensation, as shown by X. Qu et al. [271]. Poly(ionic liquid) or PIL hydrogels, described by X. Qu, are formed by copolymerizing ionic liquids with organic monomers, inherently high ionic conductivity, chemical stability, and biocompatibility, while demonstrating excellent fluid retention and mitigating IL leakage [271].

Ionic hydrogels and ionogels may function as soft ionic conductors and electrodes in flexible and wearable TENGs [273,274,275]. TENGs utilizing these materials harvest energy generated by the migration of ions under mechanical motion [275,276]. For example, in the study conducted by S. Xie, the mobility of internal anions and cations within the hydrogel skeleton drives the power generation performance [276]. The presence of dissolved electrolytes suggested by Y. Wang, such as inorganic salts, acids, or amphoteric ions, provides free ions within the hydrogel’s aqueous environment, enabling directional movement and electrical current conduction under potential difference [277]. Specific ionic liquid components, such as 1-ethyl-3-methylimidazolium dicyanamide ([EMI][DCA]) or choline-based bio-ionic liquids, have been employed, some demonstrating biocompatibility according to X. Qu et al. and I. Noshadi et al. [271,278]. Choline-based bio-ionic liquid conjugated hydrogels exhibited remarkably in vitro and in vivo biocompatibility as mentioned by I. Noshadi’s work, supporting the growth and function of primary cells [278]. The triboelectric performance of these materials is shaped by their intrinsic characteristics, as shown in Table 14.

As soft, flexible materials, they are well-suited for wearable applications and interfaces with the human body [270,275]. The tunable conductivity is a crucial property outlined by J. Yin and P. Lu’s studies, achievable by incorporating conductive additives like carbon or metal nanomaterials and conductive polymers, which create conductive pathways and accelerate ion transport [282,283]. As highlighted in the reviewed studies, MXene nanosheets in PVA hydrogels significantly improved conductivity and output performance, creating structures resembling water-filled microchannels that facilitate positive ion transport [282,283,284]. The mechanical properties, including stretchability and toughness, are also vital for device durability and performance under strain [271,275,277,285]. Evidence suggests that covalent crosslinking and synergistic interactions between components, such as electrostatic interactions and hydrophobic forces, can enhance mechanical strength and stability [271,286,287,288,289,290]. Therefore, according to Y. Wu et al., the relationship between crosslink density and ion mobility impacts ionic conductivity and device output [290].

Within the scope of additive manufacturing, particularly direct writing and 3D printing, it is an effective tool for structuring ionic liquid-based hydrogels and ionogels [270]. This enables the production of conductive structures in various forms and the creation of customized scaffolds as outlined by K. Sheikh, which is advantageous over regular hydrogels for certain applications due to fluid properties [270]. The use of ILs in 3D printing provides materials with improved mechanical strength, conductivity, and adaptability [270]. As evidenced by K. Sheikh’s research, biofunctional 3D-printable hydrogel inks have also been developed [270]. AM allows for precise control over morphology and spatial arrangement, expanding the potential uses of these materials [270]. Studies such as Y. Wang et al. mixed 3D printing techniques integrating composite structures and ionic hydrogels to create ultraflexible power generation devices [277].

The utilization of ionic hydrogel-based TENGs brings forth distinct advantages, such as their flexibility, high conductivity, exceptional biocompatibility, and transparency, making them ideal for wearable human–machine interfaces (HMIs) and implantable applications [274,275,278,283]. S. Liang et al.’s study enables self-powered systems by harvesting energy from mechanical motion [275]. Despite these advantages, critical hurdles remain, such as optimizing material composition and composites, enhancing thermoelectric performance and stability, miniaturization, and integration with wireless communication protocols [291]. Despite its potential for applications detecting human pulses and movements, some devices may require supplementary amplification circuits [257,291]. Maintaining structural integrity and preventing IL leakage can also be challenges, addressed partly by PILs and careful fabrication [261,277].

#### 3.3.3. Conductive Polymers (PEDOT:PSS)

Poly(3,4-ethylenedioxythiophene):poly(styrene sulfonate) or PEDOT:PSS is a prominent intrinsically conductive polymer widely investigated for applications in organic electronics and bioelectronics [292,293,294]. One frequently adopted method for its preparation utilizes the oxidative polymerization of the 3,4-ethylenedioxythiophene (EDOT) monomer in an aqueous dispersion using poly(styrene sulfonate) (PSS) as a template and charge-balancing counterion, which also helps disperse the conducting PEDOT chains in water [293,294,295]. Assessed academic sources indicate that PEDOT:PSS is commercially available as a water-soluble polyelectrolyte system, valued for its high electrical conductivity, good film-forming properties, excellent stability, and high visible light transparency [293,294,295,296,297]. Moreover, it demonstrates favorable biocompatibility, making it suitable for bioelectronic and neuroelectronic applications [292,294,298,299,300,301].

G. Li and G.B. Tseghai point out that pristine PEDOT:PSS tends to exhibit brittleness and rigidity, which constrains its application in flexible and stretchable electronics subjected to mechanical stress [292,302]. Still, its mechanical performance can be greatly enhanced by incorporating it into composites with elastomeric polymers such as polyurethane [296,302,303,304]. Studies conducted by S. Guzzo, Y. Kim, and Y. Jo showed that PEDOT:PSS also exhibits mixed ionic–electronic conduction [299,305,306].

PEDOT:PSS material aligns with a lot of AM techniques, enabling the fabrication of custom structures and patterns [301,307,308,309]. Methods mentioned in the literature include inkjet printing [295,296,299], screen printing [261,262,298,310], direct ink writing [306,311], electrospinning [297], wet-spinning [289], 3D printing [298,299,301,309], and electrohydrodynamic (EHD) jet printing [297]. As A.I. Hofmann’s work suggests, AM techniques facilitate the control of material deposition to create specific structures [312], including porous scaffolds [301] and fine patterns (EHD jet printing demonstrated line widths as low as 20 µm [297]). Still, the ability to achieve micron-level precision can be challenging with some techniques like conventional 3D printing and screen printing [297].

The electrical conductivity of PEDOT:PSS material plays an important role in shaping overall device performance, being significantly influenced by processing methods, additives, and morphology [296,303,313]. Techniques noted in the academic literature like secondary doping with polar solvents (e.g., dimethyl sulfoxide–DMSO and ethylene glycol–EG) [296,297,302,303], incorporating nanofillers (e.g., SnO_2_ nanoparticles and carbon nanotubes) [309,313], or post-treatments can enhance conductivity, often by inducing conformational changes in the PEDOT chains or affecting phase segregation [296,305,309]. The surface morphology and structure, controllable through AM, play a role in how the material interacts and transfers charge, relevant for applications like neural interfaces by M. Bianchi, where nanotopography is explored [314] or in porous electrodes by N. Calabia Gascón, where impregnation and internal electrical properties are key [315].

#### 3.3.4. Comparative Assessment of AM Integration Potential for Advanced Biocompatible Materials and Enhancements

MXenes appear to be highly suitable for AM-enabled TENGs, demonstrating an excellent combination of inherent high electrical conductivity, tunable surface chemistry and electronegativity, and strong compatibility with 3D printing and ink-based AM processes. They show clear advantages in enhancing charge density and overall electrical output, and their biocompatibility is promising and tunable, though oxidation remains a key limitation requiring mitigation.

Ionic liquids and hydrogels are exceptionally well-suited for AM-enhanced TENGs, particularly for wearable and implantable applications requiring conformity to the human body. Their inherent high biocompatibility (e.g., choline-based bio-ionic liquids showing in vitro and in vivo compatibility), flexibility, and high ionic conductivity make them ideal for self-powered human–machine interfaces. AM techniques like 3D printing and direct writing enable precise control over their soft, porous, and stretchable architectures. While their TENG performance relies on ion migration, challenges include preventing IL leakage and ensuring optimal conductivity compared to traditional metal electrodes.

PEDOT:PSS is highly adaptable for AM-enabled TENGs, especially as a versatile conductive component or electrode layer. Its wide compatibility with numerous printing techniques (e.g., EHD jet printing), favorable biocompatibility, and tunable electrical conductivity are significant strengths. It excels at creating integrated conductive patterns and porous structures. The main limitation is its inherent brittleness in pristine form and challenges with long-term in vivo stability under dynamic physiological conditions, necessitating composite formation and careful surface engineering for durability.

Overall, MXenes offer the most direct and highest reported TENG performance enhancement through AM (Table 15). Ionic liquids and hydrogels excel in biocompatibility and flexibility for direct biological interfaces. PEDOT:PSS provides unmatched AM versatility for conductive components. The strategies of porous structure design and surface modification are integral and indispensable enhancement approaches across all three material classes, consistently improving effective contact area, charge transfer, and device longevity when combined with AM.

## 4. Surface Texture and Porosity Engineering for Enhanced Triboelectric Performance

Tailoring the surface texture and porosity of biocompatible materials has been shown to significantly enhance triboelectric performance. Micro- and nanostructures can increase contact area and charge density, while porous architectures may improve flexibility and energy conversion efficiency. The integration of AM methods facilitates the creation of these patterns. Consequently, recent studies have begun to focus on critical modifications aimed at developing high-performance wearable and implantable triboelectric devices.

### 4.1. Surface Roughness and Porosity Control of PDMS

Various methods reported in the literature are used to control the surface texture of PDMS to enhance its triboelectric properties. Hierarchical and gradient surface textures in PDMS are primarily achieved through soft lithography, replica molding, and sacrificial template methods, which generate patterns such as micropillars or controlled porosity [28,316,317,318]. Direct ink writing allows tunable pore sizes (~330 µm), while plasma treatments further refine surface chemistry and roughness [319,320,321]. These modifications significantly enhance the effective contact area, improving triboelectric output, e.g., dual-embossed PDMS films increased voltage from 57 V (flat) to 207 V [10,46].

Soft lithography and molding techniques are standard approaches for creating specific patterns like micropillars, pores, or dimples [8,322,323]. Surface modifications mentioned by S. Vlassov et al., such as plasma treatment, can alter both chemistry and roughness; oxygen plasma treatment has been shown to result in comparatively smoother PDMS surfaces with an average roughness of 0.3 nm compared to untreated surfaces at 0.6 nm [38].

**Figure 3:**
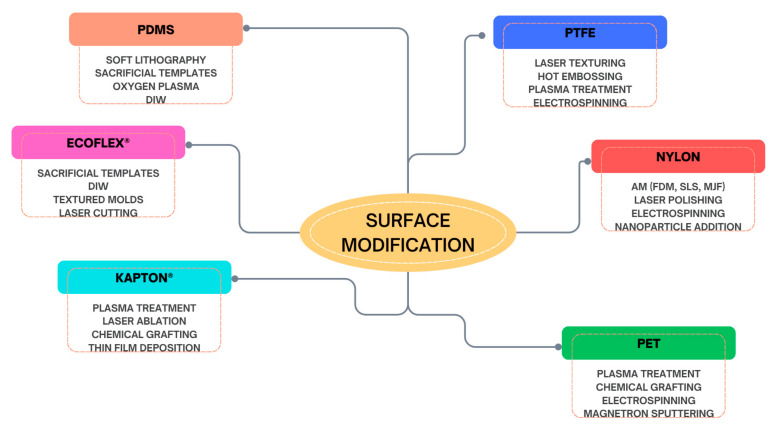
Overview of surface modification methods for selected synthetic polymers.

Chemical grafting combined with microfluidic gradient generators and photopolymerization, as described by B. Zhou, allows for the engineering of surface roughness gradients on PDMS, achieving average roughness values ranging from approximately 2.6 nm to 163.6 nm depending on monomer concentration [37]. According to A. Tropmann, incorporating particles like PTFE followed by plasma etching can create multiscale roughness, leading to superhydrophobic surfaces with contact angles over 160° [39]. To illustrate the range of surface modification strategies applied, Figure 3 provides a visual overview of the techniques used across the studied synthetic polymers.

Additive manufacturing, particularly direct ink writing, offers routes to fabricate porous PDMS structures with controlled architectures [41,42]. Porosity can be generated by removing components from printed ink, resulting in micron-sized pores; for example, R. Woo et al. mentioned diameters ranging from 0.8 to 7.5 µm and an average of 2.4 µm [43]. Sacrificial mold 3D printing methods can also be used to create porous PDMS scaffolds with specific pore characteristics, as shown by H. Montazerian et al. and R. Ariati et al. [46,50]. Lubricant-infused 3D-printed molds (OLIMs) developed by M. Villegas et al. allow the casting of PDMS with markedly smoother channels, decreasing surface roughness from approximately 2 µm to 0.2 µm [319].

Surface texturing, particularly the construction of micro/nanoscale structures, is a pivotal approach to obtaining functional surfaces, including superhydrophobic properties by trapping air, as shown by J. Liu et al. [36]. For TENGs, as D. Choi mentions, increasing the effective surface contact area through surface embossing [35] or incorporating porous structures (J. Li et al.) can significantly enhance triboelectric output [33]. Highly surface-embossed PDMS configurations, achieved by replicating nanoscale structures, increased the surface contact areas, like in D. Choi’s study, contributing to enhanced surface charge density and electrostatic induction [35]. Porous PDMS films have demonstrated, in J. Li’s study, a significantly higher generated voltage (17 V) compared to nonporous PDMS films (5 V), attributed to increased effective area and capacitance [33]. Nanopatterned PDMS surfaces reported by M. Ji et al., created by replica molding, allow for the mapping of tribocharges using techniques like Kelvin Probe Force Microscopy (KPFM) and Electrostatic Force Microscopy (EFM), revealing insights into charge distribution and stability [318,324]. While surface texture can strongly influence tribological properties like friction [322,323], the relationship between surface roughness and the magnitude of charge transfer or resulting electrochemical activity is complex and is not necessarily directly proportional at the nanoscale, as shown by J. Zhang et al. [325].

Despite inherent challenges like hydrophobic recovery and oxidative degradation under physiological fluid exposure [36,321,326], the long-term tribocharge stability of engineered polydimethylsiloxane surfaces have been confirmed for over 14 days [324], with stability further enhanced by covalent functionalization with zwitterionic polymers that maintain hydrophilicity for over 74 days in wet conditions [36,320].

### 4.2. Surface Roughness and Porosity Control of PTFE

Laser texturing and hot embossing produce hierarchical micro/nanostructures on PTFE, such as groove or cotton-like morphologies, which amplify surface area and charge density [35,43,327]. Plasma treatment complements this by adjusting surface chemistry for better adhesion and hydrophilicity [16,26]. TENGs using microgrooved PTFE arrays show over three-fold increases in voltage and current compared to unmodified PTFE, highlighting the direct correlation between textural hierarchy and output [104].

Laser texturing using different laser sources (e.g., UV and CO_2_) can create hierarchical micro- and nanostructures according to A. Riveiro et al. [53]. These structures can appear as spongy [53], cotton-like [53], hairy-like [53], or groove patterns [53,67], increasing surface roughness [26,53,66,67]. Laser texturing can introduce significant porosity and air pockets [53], often resulting in superhydrophobic surfaces, as mentioned in F. Chen and A. Riveiro’s research [53,66]. Plasma treatments, such as cold plasma or dielectric barrier discharge, can also roughen the surface at micro- and nanoscales [66,69,328] and alter surface chemistry by introducing functional groups [57,66,69], affecting wettability [66].

AM techniques like DIW and vat photopolymerization allow for the fabrication of 3D PTFE microstructures with controlled porosity and complex geometries [42,58,60,61]. The sintering process in these methods reported by Y. Zhang et al. and Z. Jiang et al. fuses PTFE nanoparticles, yielding structures with specific porosity depending on parameters like water content in the ink [58,60].

Surface morphology significantly influences triboelectric charging. Increased surface roughness and hierarchical structures created by texturing methods enhance the effective contact area between materials during triboelectric contact [10,66,328], which is critical for improving the charge transfer and electrical output of TENGs [10,26]. For example, B. Dudem used microgroove array (MGA)-textured PTFE as opposed to pristine PTFE, which resulted in a notable increase in open-circuit voltage (from ~85 V to up to ~320 V) and short-circuit current (from ~3 μA to up to ~15 μA) [26]. Similarly, P. White uses electrospun porous PTFE/PVDF fibers with increased surface roughness that demonstrated significantly higher voltage and current outputs compared to pure PVDF fibers [21]. Surface defects mentioned by G. Fatti et al. and F. Chen et al., such as fluorine vacancies introduced during processing like laser ablation or plasma treatment, can also create trap states that facilitate charge acquisition and retention [63,66].

While PTFE exhibits chemical inertness, its long-term stability under physiological conditions and mechanical stress can be notably improved; for instance, laser-textured surfaces remain unaltered after 2 h in strong acid (pH 1) or alkali (pH 14) solutions and demonstrate wettability maintenance after 50 finger-wipe cycles (Riveiro et al.) [53], with further long-term stability of hydrophilicity and bonding strengths achieved through femtosecond laser texturing-assisted cold plasma modification (Chen et al.) [66].

For biocompatible applications, modified surface morphology and chemistry can improve cell adhesion or prevent unwanted cell growth, which is crucial for devices like implants or stents [53,57,58,59,60]. AM methods facilitate the creation of customized structures for these biomedical applications [5,58,60,61,160].

### 4.3. Surface Roughness and Porosity Control of Ecoflex^®^

Gradient and porous architectures in Ecoflex^®^ are fabricated using sacrificial templates (e.g., sugar or NaCl) and DIW printing, yielding controlled pore networks (~330 µm) [53,329,330]. Advanced strategies like gradient interpenetrating polymer networks (g-IPNs) further optimize mechanical durability and triboelectric enhancement [66]. These multiscale structures elevate output voltage (e.g., 30 V vs. 23 V for nonporous) and improve device resilience under strain, ideal for wearable and stretchable TENGs [53,161].

This involves mixing Ecoflex^®^ with particles such as granulated brown sugar [90,93,331] or sodium chloride (NaCl) [327]. After the Ecoflex^®^ is cured, the template particles are dissolved in a solvent like water, leaving behind a porous structure that replicates the inverse of the template geometry [90,93,327]. Using NaCl templates, porous structures with pore diameters of approximately 330 µm have been fabricated by Q.L. Goh et al. [327]. Alternatively, controlled porosity can be achieved as shown by Z. Song et al. through physical methods, such as using a laser cutting machine to create precise patterns of holes, like a circular array of 2 mm diameter holes in an Ecoflex^®^ layer [77]. The presence of these pores and internal air gaps in Ecoflex^®^ enhances charge generation and transfer, contributing positively to TENG output [74,327]. For instance, porous Ecoflex^®^ TENGs have demonstrated in X. Wang et al.’s study a higher voltage output (30 V) compared to their nonporous counterparts (23 V) [74]. A challenge associated with introducing porosity, reported by J. Zhang et al., is ensuring sufficient mechanical robustness, as excessive void volume can compromise the material’s structural integrity [332].

Surface roughness and microstructures are commonly imparted to Ecoflex^®^ surfaces using textured molds or templates during the curing process [80,81,333,334]. This can involve casting Ecoflex^®^ against surfaces created by 3D printing with defined patterns as shown by J. Zou et al. and K.A. Eltoukhy [80,81], using textured materials like sandpaper to yield random “frosted microstructures” consisting of interconnected protrusions and depressions (X. Wang et al.) or even employing natural structures such as banana leaves to replicate bionic parallel vein-like microstructures, as shown by B. Yin et al. [333,334]. Micropatterning techniques reported by H. Xiang et al. are also utilized to create defined textures on Ecoflex^®^ for TENG applications [4]. These surface modifications, including rough microstructures, significantly increase the effective contact–separation area between the Ecoflex^®^ layer and the counter-triboelectric material, as shown by X. Wang et al. [333]. This leads to enhanced charge transfer and improves electrical output performance [80,329,332,333]. Structured surfaces in composites, such as rough microstructures in MXene/Ecoflex^®^, have been associated in S. Yang et al.’s study with very high TENG outputs, reportedly reaching 790 V open-circuit voltage, 183 µA short-circuit current, and 9.5 W/m^2^ power density [15]. The successful control of both porosity and surface texture makes Ecoflex^®,^ as outlined by X. Wang, a highly suitable material for developing high-performance, biocompatible TENGs [74].

Engineered Ecoflex^®^ surfaces exhibit robust long-term stability under physiological-like conditions, exemplified by nanocomposite embedding strategies, such as multi-walled carbon nanotubes (MWCNTs), which enable strain sensors to endure over 33,000 stretch-and-release cycles at 20% strain [14], and by its inherent hydrophobic properties, demonstrating resistance to salt-aging over 7 days at 80 °C, as shown by Truong et al. [335].

### 4.4. Surface Roughness and Porosity Control of Kapton^®^

Surface modification of Kapton^®^ via CF_4_/CH_4_/Ar plasma increases roughness and lowers surface potential energy, enhancing triboelectric charge generation [110,150]. Trifluoromethyl (-CF_3_) grafting further augments electron-withdrawing capacity, resulting in 4–5× higher output than untreated films [97]. Reduced graphene oxide fillers also act as electron traps, boosting power density up to 30-fold [97]. The pristine Kapton^®^ surface is typically smooth and flat, with reported roughness values below 10 nm [336], including one measurement around 1.60 nm [100] and another at 1.9 nm [337].

To enhance Kapton’s surface properties for applications including triboelectric energy harvesting, various surface modification techniques have been investigated. Plasma treatments are a common approach, as mentioned in R. Seeböck et al.’s study, leading to surface roughening visible even to the naked eye [336]. Methods like Ar + O_2_ plasma treatment can evolve the surface morphology from a dense nano-feather structure to nano-tussock/nano-fibrils with increased treatment time, resulting in significantly higher roughness (58.4–396.0 nm compared to 1.9 nm for untreated) according to H.C. Barshilia et al., and creating void spaces [337]. Dielectric barrier discharge (DBD) plasma also causes surface roughening through etching, forming nanostructures [95,336]. Other modifications include chemical treatments using alkali solutions or silanes to improve hydrophilicity and adhesion [338], laser texturing to improve wettability [103,339], and ion implantation [340]. Depositing thin films or composite layers, such as titania (I. Gouzman et al.), Al_2_O_3_ nanocomposites (D. Jiang et al.), ZnO (A. Bardakas et al.), or rGO@MnO_2_ (M.A. Alouani), on Kapton^®^ substrates also alters the surface morphology and composition [96,109,341,342]. AO erosion, a factor in space environments, can also create a rough, carpet-like morphology on Kapton^®^ [96,108,343].

While T. Rodrigues-Marinho et al. notes that a smooth surface on commercial Kapton^®^ can decrease triboelectric performance [110], increased surface roughness is generally understood to provide a larger effective contact area in TENGs [125,340], which can lead to more efficient charge separation and an increased total amount of charge transfer, resulting in higher output voltage and current, as shown by B. Sun et al. [125]. Modified surface chemistry through techniques like functional group introduction or the incorporation of conductive or high dielectric nanoparticles or 2D materials can further tune the charge density, dielectric constant, and charge storage capacity of the friction layer, enhancing the triboelectric nanogenerator’s performance [5,11,104,125]. Surface modifications also improve the adhesion and printability of other functional materials on Kapton^®^, which is crucial for fabricating flexible electronic devices and sensors, as mentioned by Y. Fang et al. and I.I. Labiano et al. [101,344].

In the context of biocompatible AM systems, Kapton’s biocompatibility [102] makes it a suitable foundation, as shown by M.A. Signore et al. Although the direct AM of Kapton^®^ films with controlled surface texture specifically for triboelectric applications is not explicitly detailed in the recent studies, Kapton^®^ is successfully used as a flexible substrate for various processes akin to AM or patterning, such as the deposition of thin films [101,107,345] and the fabrication of micro-devices using multi-step processes including sputtering, photolithography, and electrodeposition, according to M. Khelifa et al. [100]. Furthermore, bio-enabled surface modification methods on Kapton^®^ enable excellent printability for techniques like inkjet printing, allowing the fabrication of patterned functional layers for flexible electronics and wearable sensors [101,342]. This demonstrates that surface engineering on Kapton^®^ is key to integrating it into advanced patterned and potentially AM-related structures for enhanced performance in applications requiring charge generation or sensing, including those involving flexible and biocompatible interfaces.

Engineered Kapton^®^ surfaces by J. Guo et al. demonstrated enhanced stability for biomedical applications, such as neuronal cell biosensors, where inkjet printing graphene ensures biocompatibility with approximately 85% cell viability after 72 h under physiological conditions and exhibits negligible changes in conductivity over time [346].

### 4.5. Surface Roughness and Porosity Control of Nylon

Nylon is a prominent engineering thermoplastic widely used in AM, particularly in techniques like FDM and SLS, for fabricating parts with complex geometries [1,121,132]. These AM processes inherently influence the surface characteristics of nylon components. Comparative studies such as M. Arioli et al. show that different AM methods result in varying surface roughness; for instance, Multi Jet Fusion (MJF) parts can exhibit improved surface roughness compared to SLS counterparts, attributed to distinct heating effects [1]. FDM-printed nylon–carbon fiber composites also show variations in surface roughness according to S. Hartomacioğlu et al. [137].

Post-processing techniques offer further control over nylon’s surface texture. Laser polishing can alleviate asperities on 3D-printed Nylon-6 surfaces, demonstrating a 20.2% reduction in roughness as mentioned by R.T. Mushtaq et al. [136]. Other methods include thermal and chemical treatments (M.S. Saharudin et al.), CNC machining (M.S. Saharudin et al.), polishing (P. Le Bars et al.), and atmospheric plasma treatment, which improves surface wettability and can influence roughness, as shown by M. Rodríguez et al. [118,122,347]. Beyond solid structures, electrospinning is used to create porous nylon nanofiber textiles (P. Čapková et al.), often with fiber diameters around 100 nm (O.Y. Antonova et al.), which can serve as scaffolds or membranes [128,129]. The arrangement and homogeneity of fibers mentioned by P. Čapková in these porous structures can be influenced by processing parameters like electrode distance [129]. Macroscopic nylon textiles can exhibit significant roughness, recorded at 90.6 ± 0.3 µm according to N. Matijaković Mlinarić, for one type [131]. Electrospun-aligned nylon nanofibers exhibit high surface area and tunable morphology, ideal for energy harvesting [11,128]. Hierarchical structures formed via CNT grafting increase roughness and the effective contact area, enhancing voltage and current output [33,104,125]. Nanoparticle fillers (e.g., h-BN and graphene) improve wear resistance and friction properties through tribological film formation [117,125,348,349].

Nylon is known to be on the positively charged side of the triboelectric series [1,121]. As evidenced in the findings of M. Arioli and N. Wang, when rubbed against other materials, nylon is more likely to lose electrons and become positively charged [1,52]. This property makes nylon a potential candidate as a tribopositive material in TENGs [1]. The magnitude of charge transfer in contact electrification depends on the relative position of the contacting materials in the triboelectric series, as reported by N. Wang et al. [52]. Surface properties, including roughness and hydrophilicity, are critical in biomedical applications, influencing factors such as microbial colonization on prostheses or cell adhesion on tissue engineering scaffolds [122,129].

Despite nylon’s susceptibility to moisture sensitivity and potential degradation under certain long-term exposures [1,350], its inherent biocompatibility and chemical stability are leveraged for biomedical applications [1,350], with strategies such as silica nanoparticle–Nylon 6 composite formation demonstrating colloidal stability for at least 7 months in storage and 10 days in fetal bovine serum enhancing durability for drug delivery systems [130].

While the recent studies discuss methods to control nylon’s surface texture through AM and post-processing and highlight nylon’s triboelectric properties for TENGs and its relevance in biomedical applications, most of the literature does not explicitly detail how specific, controlled changes in surface roughness or porosity in nylon directly influence or quantify changes in its triboelectric charging behavior (e.g., contact electrification magnitude or charge retention) within the context of additively manufactured biocompatible systems. Challenges in utilizing nylon for such applications include improving surface adhesion and precisely controlling surface properties for optimal performance and safety [118,122,131].

### 4.6. Surface Roughness and Porosity Control of PET

Surface modification techniques are employed to tailor PET’s surface roughness and texture. O_2_ or CF_4_/CH_4_/Ar plasma treatments on PET generate hydroxyl groups and surface roughness, elevating triboelectric performance [143,150,351]. Molecular self-assembly introduces amide groups for enhanced hydrogen bonding and electron donation [104]. Coating PET with BNNSs (2D boron nitride nanosheets) significantly increases the dielectric constant, enabling up to 70 × improvement in output power [5,104,150].

Plasma treatment, using gases such as air, oxygen, or argon, is effective in increasing PET surface roughness and introducing new functional groups containing oxygen and nitrogen [145,150,352,353]. Air plasma treatment, mentioned in L. Yang et al.’s study, has been shown to increase the arithmetic average roughness (R_a_) of PET films from approximately 2.0 nm to values ranging from 10.9 to 14.8 nm, depending on the exposure area [352]. Similarly, V.N. Szafran et al.’s study mentioned that the root-mean-square roughness (S_q_) increased from about 2 nm for unmodified PET to roughly 4 nm after air plasma activation [143]. This increase in roughness is attributed to etching by high-energy active plasma particles, as shown by L. Xia et al., creating concave–convex structures and enhancing the surface area [353]. According to S.H. Shin et al., the plasma treatment also forms reactive sites, such as hydroxyl groups, enabling subsequent chemical functionalization [151,152].

Chemical functionalization, including grafting copolymers or attaching specific molecules like halogens and amines, can dramatically alter PET’s surface properties and triboelectric behavior [141,151,152]. Grafting can change a hydrophobic PET surface into an amphiphilic one, improving biocompatibility, as shown by Y. Jiang et al. [141]. Atomic-level chemical functionalization allows for tuning triboelectric properties, achieving a wide spectrum of polarities on a single PET material, as shown by S.H. Shin et al. [152]. Such modifications have resulted in significantly enhanced TENG output power, reported by S.H. Shin to be around four orders of magnitude higher compared to non-functionalized surfaces [151]. For instance, a functionalized PET contact pair yielded a maximum open-circuit voltage of approximately 330 V and a short-circuit current density of about 270 mA/m^2^ (S.H. Shin et al.), with a maximum power density of approximately 55 W/m^2^ achieved with an optimal pair, as shown by S.H. Shin et al. [151,152]. The triboelectric charge density for a PET and blend pair was measured at 390 nC/m^2^, as reported by P. Slobodian et al. [149].

Furthermore, coating and deposition methods, such as magnetron sputtering, can modify the roughness of PET substrates, creating thin films with R_a_ values between 3 and 9 nm (A. Marciel et al.) or significantly rougher surfaces (~140–203 nm R_a_) on nanofiber-modified PET fabrics (H. Ye et al.) [158,354]. While Langmuir–Blodgett (LB) monolayer deposition on plasma-treated PET can decrease roughness (S_q_ down to ~0.8 nm), as mentioned by K. Szafran [143], increased surface roughness generally leads to higher surface charge generation, enhancing the efficiency of nanogenerators [149,150,353]. Coarser surfaces reported in P. Slobodian et al.’s study are associated with higher surface charges [149]. Structuring PET into porous nanofiber networks via electrospinning, like L.W. Lai et al.’s research, also creates materials with a large surface-to-volume ratio suitable for TENGs [148].

PET surfaces are initially prone to hydrolytic degradation and poor biocompatibility under physiological conditions, potentially leading to material deterioration and reduced performance [143,355]. However, surface modification via oxygen plasma treatment followed by chemical functionalization has demonstrably improved stability, enabling PET-based triboelectric nanogenerators to maintain consistent output performance over 7200 cycles for 2 h and repeatedly over 4 weeks without degradation, primarily due to the formation of strong covalent bonds on the modified surface [151].

These surface engineering approaches address the inherent limitations of PET, improving properties like wettability and biocompatibility [141,143]. The ability to control surface roughness and chemistry on PET substrates is fundamental for optimizing their performance in biocompatible triboelectric systems, such as wearable sensors and energy harvesters [145,148,356].

### 4.7. Surface Roughness and Porosity Control of Graphene

Surface texture and porosity in graphene can be engineered through various methods relevant to AM and composite fabrication [167,182]. Laser-induced graphene fabrication is a facile, single-step approach to create 3D porous graphene structures on surfaces without the use of solvents or chemical reagents [78,173,357]. This method results in a porous microstructure containing carbon nanofibers and micropores, which serve as active triboelectric materials [178,357]. LIG-based triboelectric nanogenerators (TENGs) demonstrated high power output, reaching up to 0.76 W m^−2^, and can improve the surface potential by acting as a charge-trapping intercalation layer [5]. Additionally, crumpled graphene (CG), formed by transferring graphene onto prestrained tapes, exhibits high surface roughness and an effective contact area, which significantly enhances TENG output performance by increasing charge density, output voltage, and current density [4,358].

Techniques include fabricating 2D sheets (H. Mohammed et al.), stacking layers (graphene nanosheets, GNSs, can range from monolayer to 100 nm thick—B. Xie et al.), creating 3D porous structures like aerogels (K. Tadyszak et al.) or laser-induced graphene (LIG—P. Xue et al. and S.P. Singh et al.) or forming wrinkled/crumpled topographies (P.Y. Chen et al.) and composite nanofibers (Y. Jiao et al.) [163,164,172,173,357,358,359,360]. LIG is a one-step, solvent-free method creating conformal 3D porous graphene structures on surfaces [357,359].

Engineering surface texture significantly impacts triboelectric performance. The large specific surface area of graphene [42,75,164,168,169,361] is key to facilitating charge transfer during contact electrification [174,175]. High electron mobility [166,169,175,361] and charge storage capabilities (S.M. Kim) further enhance triboelectric output [174]. Specific surface textures, such as the carbon nanofibers (250–750 nm diameter) and micropores (1–25 μm) observed in LIG films by S.P. Singh [357], influence the contact mechanics and charge transfer dynamics [357,359]. These features can potentially reduce the effective contact area and influence abrasive wear according to P. Xue [359]. Aligning graphene sheets within polymer matrices, as in PDMS composites, can also improve TENG performance [175,180]. Quantitative results from PVDF–graphene nanofibers reported by Y. Jiao et al. demonstrate enhanced TENG output, achieving an open-circuit voltage of 245 V, a short-circuit current of 24 µA, and a charge transfer of 80.2 nC [172].

While graphene surfaces exhibit excellent mechanical wear resistance, with laser-induced graphene films demonstrating stable performance over 20,000 cycles [359], their long-term stability under physiological conditions is a critical concern due to potential degradation and cytotoxicity [165,169,362,363]. To address this, surface functionalization strategies like PEGylation have proven effective in improving biocompatibility and facilitating gradual in vivo excretion over several months [164,169,363].

Surface engineering, including controlling texture, is essential for designing safe and effective interfaces; for example, LIG texture has been correlated with enhanced anti-biofilm properties [357]. Therefore, tailoring surface texture for optimal triboelectric function in biocompatible systems requires the careful consideration of the material’s biological response [165,167].

### 4.8. Surface Roughness and Porosity Control of CNTs

The electrophoretic deposition (EPD) of CNTs is a suitable technique to coat metallic surfaces with predefined roughness in a reproducible manner, creating textured layers [1]. This process leads to a mixture of rolling motion on surfaces and particle degradation, including the formation of nanocrystalline graphitic layers [364]. MWCNT coatings, applied via EPD, have been shown to considerably decrease the friction coefficient of steel surfaces from 0.7 to 0.2 [364]. Furthermore, high-performance TENGs have been developed using aligned carbon nanotubes, which leads to enhanced triboelectric output [16,365]. Carbon Nanotubes, known for their exceptional mechanical, electrical, and thermal properties, alongside a high aspect ratio and large specific surface area, are promising fillers for modifying the surface texture and bulk properties of composites [185,186,188,189,191,192,193,366,367,368]. These materials consist of rolled graphene sheets with hexagonal structures, existing as single-walled (SWCNTs) or multi-walled (MWCNTs) forms [185,187,189].

A significant challenge in integrating CNTs into matrices, particularly polymers for AM, is achieving uniform dispersion due to strong Van der Waals forces and their high surface area, which promotes agglomeration [192,197,368,369]. Agglomeration can lead to non-uniform distribution and inconsistent surface properties in the resulting composite. To overcome this, various techniques are employed, including ultrasonication and chemical functionalization, which introduce active surface groups (e.g., carboxyl or hydroxyl) to enhance compatibility and stability within the matrix [189,192,200,368,369]. Surface modification and textural adjustments according to S. Abreu et al.’s study can also influence CNT size and facilitate the incorporation of biocompatible groups [369].

The surface morphology and arrangement of CNTs within composites significantly influence triboelectric and related electrical properties. For instance, in cellulose–nanocarbon composites, the specific surface area of the filler, its nanoscale dispersion, and the matrix porosity have a stronger influence on the triboelectric output voltage than electrical conductivity alone, as reported by J. González et al. [196]. Engineered CNT structures, such as aligned arrays or sheets [186,190,366,367], 3D networks (X. Huang et al.), or incorporation into porous scaffolds [196,370,371], can further tailor surface interactions and bulk properties. For example, in X. Huang et al.’s research, three-dimensional CNT networks effectively reduce ion diffusion paths and enhance electrolyte penetration in electrochemical applications [370]. Surface roughness, influenced by CNT incorporation, has also been shown by Q. Cao et al. to affect electrical interface properties and catalytic activity, with rougher surfaces potentially yielding higher current density and sensitivity [372]. Nitrogen-doped CNTs (N-CNTs) can exhibit anomalous piezoelectric properties, where the electrical response to mechanical deformation is linked to defectiveness, geometric parameters (e.g., aspect ratio), and the structural perfection of sidewalls, as shown by M.V. Il’ina et al. [195].

In the context of AM techniques like FFF, CNTs are added to polymers such as PLA and HDPE to enhance electrical conductivity, achieving low percolation thresholds [197,198]. The inclusion of CNTs can also impact the surface roughness of printed parts, with studies suggesting that increased CNT content can lead to a reduction in surface roughness in specific directions [373,374,375]. This modification of the surface texture during the printing process is directly relevant to the triboelectric performance of the final part.

While CNTs demonstrate significant mechanical stability, with 3D-printed nanocomposites showing no considerable change after 1000 bending cycles [191], their dispersibility and long-term behavior under physiological fluid exposure are critical considerations due to potential interactions and toxicity [194,376]. To address these challenges and improve biocompatibility, surface functionalization strategies such as PEGylation are employed, as seen in studies demonstrating in vitro biocompatibility with pyrene–polyethylene glycol coatings on CNTs [185,376].

**Figure 4:**
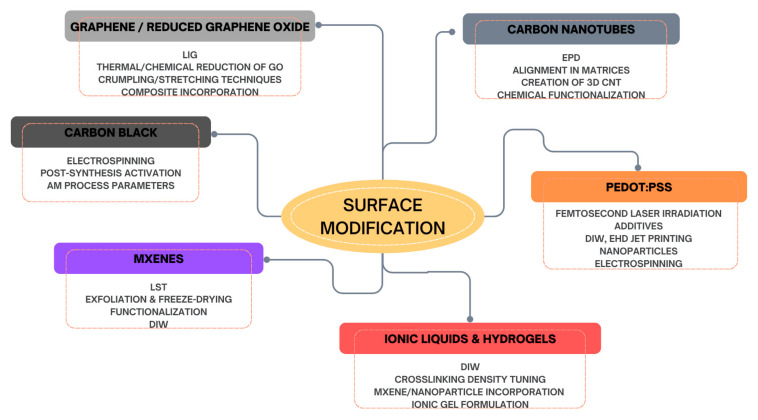
Overview of surface modification methods for carbon-based and advanced materials selected for this study.

Using surface-engineered CNTs in wearable, implantable, or AM-enabled triboelectric systems leverages their ability to form conductive networks and provide tunable surface interactions [198,368]. Advantages include improved mechanical and electrical performance and potential biocompatibility [188,193,194,199,369]. However, limitations primarily revolve around dispersion challenges and potential toxicity concerns depending on CNT type, purity, and functionalization [185,186,187,188,369]. An illustrative summary of the surface modification strategies for the carbon-based and advanced materials examined is shown in Figure 4.

### 4.9. Surface Roughness and Porosity Control of rGOs

Reduced graphene can be textured through methods like thermal reduction from graphene oxide, retaining oxygenated functional groups primarily on its edges, which facilitates surface modification [222,227]. These functional groups enable the fine-tuning of the surface chemistry for improved triboelectric properties [222]. In triboelectric nanogenerators, rGO can act as electron-trapping sites within the friction layer, leading to giant triboelectric enhancement and increased output voltage [5,173,377]. Additionally, 3D-printed porous structures fabricated using DIW with a GO gel ink, followed by thermal reduction, result in stable materials highlighting textured rGO’s potential in energy applications [226].

rGO derived from GO is a promising material for applications requiring tuned surface interactions and charge transfer, such as triboelectric devices, particularly in the context of AM and biocompatibility [213,223,226,378,379]. Unlike pristine graphene, rGO retains a certain density of residual oxygen-containing functional groups and structural defects, primarily located at the edges, which significantly modulates its electronic properties and surface chemistry [205,213,214,222]. These features are crucial for controlling surface roughness, porosity, conductivity, and ultimately, charge accumulation and dissipation in triboelectric interfaces, according to A. Smirnov et al. and d. Scarpa et al. [212,213].

Various methods are employed to engineer the surface texture and porosity of rGO. Chemical reduction in GO using agents like hydrazine hydrate is a common approach, involving the removal of hydroxyl groups and amorphization of sp^2^ carbon structures, which can influence the resulting sheet morphology and porosity [206,379,380]. P. Das. et al. shows that thermal reduction, even at low temperatures such as 50 °C, can effectively remove oxygenated functional groups and restore sp^2^ structures, altering surface properties [211]. Higher temperature thermal reduction can further eliminate residual surface oxygen as reported by K. Ge et al. [204]. Morphological analysis performed by D. Perumal et al. and P. Viprya et al. reveals that rGO typically forms crumpled thin sheets, rougher surfaces, and wave-shaped corrugated structures [206,208]. The incorporation of rGO into materials or composites can also influence surface morphology; for instance, rGO inclusion in electrospun fibers can reduce fiber diameter and potentially increase specific surface area, as shown by F.E. Che Othman et al. [381]. Specific surface areas can vary significantly depending on the preparation method, with reported values ranging from 19 m^2^/g for certain Cu/rGO composites to much higher values (e.g., up to 1780 m^2^/g) when incorporated into porous carbon matrices, demonstrating the tunability of porosity [209,217]. The removal of functional groups during reduction is often associated with an increase in porosity, as shown by P. Jagódka et al. [209].

The conductivity of rGO, enhanced by the removal of oxygen groups and the formation of interconnected carbon networks, is crucial for charge dissipation and collection in triboelectric devices, as shown by A. Smirnov et al. [212]. The presence of defects and residual oxygen groups provides active sites for surface interactions and can enhance charge carrier accumulation [205,213,382]. Surface roughness and porosity increase the effective contact area and provide pathways for electrolyte or air shuttling, influencing surface charge dynamics and overall device efficiency [378,382,383].

Reduced graphene oxide surfaces, while offering significant mechanical and tribological advantages like 51.85% wear reduction in lubricants [379] and 90% patency in tissue-engineered vessels over seven days [169], present complex long-term stability considerations under physiological conditions due to potential degradation and dose-dependent cytotoxicity [165,169,384,385]. Surface functionalization, such as PEGylation, or incorporation into stable polymer composites are vital strategies to improve their biocompatibility, degradation profile, and overall longevity in fluidic and mechanically stressed environments [164,169,219,222,384].

In the context of AM, studies like those conducted by C. Ramírez and Y. Zhao have shown that rGO can be incorporated into printable inks for technologies like direct ink writing to create structured electrodes and composites with controlled porosity and feature resolution [226,378,379]. While challenges remain in optimizing dispersion and long-term stability (M. Sarno et al.), rGO demonstrates potential for improving the mechanical properties of AM feedstocks and enabling conductive pathways [212,222,223]. Furthermore, rGO is considered less toxic than GO in some studies, suggesting its potential for biocompatible applications [206,219]. However, as mentioned by P. Wilczek, the specific surface properties and concentration of rGO can influence its biocompatibility, highlighting the need for careful surface engineering in the development of AM-based biocompatible triboelectric materials [219].

### 4.10. Surface Roughness and Porosity Control of Carbon Black

The interplay between the intrinsic textural properties of CB and the structure induced by the AM process significantly influences the performance of devices like triboelectric nanogenerators [196,213]. Carbon black can be integrated into materials using methods such as electrospinning to form composite fibers or via material extrusion-based AM into a porous polymer matrix [235,239]. This incorporation modifies the conductive network and surface characteristics of the composite [184,386]. The addition of CB to electrospun fibers has material-dependent effects on triboelectric output, decreasing it for polyurethane and polystyrene, but significantly increasing it by 260% for polycarbonate. When used in a porous polydimethylsiloxane (PDMS) matrix through AM, CB serves as a charging material for high-efficiency triboelectric sensors [226].

Carbon black, according to V.A. Drozdov et al., inherently consists of primary particles/globules (~15–300 nm) that form aggregates (~85–500 nm), which in turn link into agglomerates (~1–100 µm) [234]. The space within and between these structures, as the study mentions, creates a porous network, typically featuring meso- and macropores (~2–120 nm) [234]. Post-synthesis treatments like those outlined by Z. Jankovská et al., such as activation, can further modify CB’s porous structure and significantly increase its specific surface area (e.g., from 88 m^2^/g to 644 m^2^/g reported for different CB types) [387].

When incorporated into polymer matrices, the final composite’s structure, including its porosity and the dispersion of CB, is also affected by the AM method and process parameters (e.g., layer thickness, raster width, air gap in FDM) [246]. These parameters mentioned by J. Zhang can introduce voids and influence bonding, leading to anisotropic electrical properties [246]. J. González noted that the specific surface area and nanoscale dispersion of the filler, along with the matrix porosity and filler content, are important factors influencing the composite’s triboelectric output voltage [196]. For instance, increasing the surface roughness of a CB–silicone electrode resulted in superior TENG performance, attributed to increased contact area and enhanced electron transfer, as shown by B. Liu et al. [388]. Achieving a critical filler concentration to form conductive percolation pathways is vital for adequate charge transfer and optimizing TENG power output [82,318]. While higher concentrations generally increase conductivity (N. Vidakis et al.), exceeding an optimal level can lead to agglomeration, creating leakage paths that decrease electrical output (J. Zhang et al.) [245,246]. The effect of CB on triboelectric properties is also dependent on the specific polymer matrix used, as shown by P.K. Szewczyk et al. [239].

Carbon black exhibits notable mechanical robustness within polymer composites, with CB/PVDF flexible pressure sensors demonstrating high repeatability and reproducibility under diverse loading and vibrational conditions [389]. Furthermore, CB aggregates are remarkably stable under fluid exposure, with evidence indicating that they do not disaggregate into smaller particles in lung fluid [233]. The long-term stability and performance of CB-engineered surfaces, as seen in sensors, can be improved through optimized concentration within porous polymer matrices, leading to durability exceeding 4000 cycles for some applications [386,390,391].

The advantages of using CB include its cost-effectiveness compared to other carbon fillers like graphene or CNTs, according to Y. Tavakoli et al. [244]. Challenges involve ensuring uniform dispersion to avoid agglomeration [332], controlling anisotropic properties induced by AM processes [246,247] and carefully selecting the polymer matrix and CB content to achieve desired biocompatibility and optimal triboelectric performance [235,237,239].

### 4.11. Surface Roughness and Porosity Control of MXenes

Laser surface texturing (LST), such as creating line- and cross-textures, has been explored to improve the tribological performance of Ti_3_C_2_T_x_ MXene coatings on substrates like steel (AISI 304) and TiAl_6_V_4_ [392]. This method creates lubrication reservoirs that enhance the durability and effectiveness of the MXene coating, leading to a smooth and stable frictional evolution and reduced COFs to approximately 0.2 across all textured steel substrates [392]. Additionally, the incorporation of 2D MXene sheets into hydrogels, leveraging their negative surface charges and hydrophilicity, can enhance power generation performance in triboelectric sensors by increasing net cation flux, as seen in a polyurethane–ionic liquid composite reaching a 219 mV output [276,311,393]. MXene’s intrinsic low shear strength also contributes to friction reduction [393,394].

Analyzing the specialized literature, the surface morphology of Ti_3_C_2_T_x_ MXene, encompassing its layering, roughness, and porosity, significantly impacts its properties relevant to triboelectric applications, particularly in AM and potentially biocompatible systems. As described by Y. Li et al. and Z. Otgonbayar et al., MXenes are derived from MAX phases via selective etching, yielding structures ranging from accordion-like multilayers to delaminated few-layer or single-layer nanosheets [263,395]. Etching conditions (e.g., HF, LiF/HCl, molten salt) dictate the resulting morphology and surface terminations (–F, –OH, =O, halogens), which are mandatory for surface chemistry and electronic properties [255,258,396].

Surface morphology can be tailored through various methods. Delamination and exfoliation yield materials with expanded interlayer spacing [252,263,396]. Techniques like the addition of porous materials (F. Yue et al.) or processes such as freeze-drying in conjunction with additive manufacturing like DIW printing can create porous structures (M.L. Matias et al.), notably increasing the high specific surface area [256,261,397]. Surface roughness can be increased by incorporating materials like CNF or OMC [397,398] or through particle agglomeration during composite fabrication, as shown by Y. Gao et al. [307].

Higher surface roughness has been shown by Z. Sun et al. to result in higher output performance in TENGs [398], attributed to an increase in effective contact area and therefore surface charge density [307]. The porous nature and large surface area provide abundant active sites for interactions [256,257]. Furthermore, the interlayer distance and surface functional groups (–F, –OH, =O) govern the electronic properties, affecting conductivity and electron transfer capabilities [255,256,258,396]. Bare Ti_3_C_2_ is metallic, while functionalized derivatives like Ti_3_C_2_F_2_ and Ti_3_C_2_(OH)_2_ can be semiconductors [256,258]. Surface chemistry also affects charge distribution and the presence of locally generated dipoles [258,259]. The reported quantitative metrics include interlayer spacing around 1.02 nm for N-doped MXenes (A. Dey et al.), surface roughness values varying from 1.195 nm to 3.331 nm (F. Yue et al.) and 1.8 µm to 2.6 µm (Z. Sun et al.), and high electrical conductivity ranging from 3 Ω/sq (sheet resistance) to up to 24,000 S/cm, as shown by M. Malaki et al. and Z. Sun et al. [252,255,397,398].

MXenes face challenges in long-term stability under physiological conditions due to oxidation in oxygen-dissolved water and susceptibility to mechanical wear [268,394]. However, strategies like laser surface texturing create lubricant reservoirs that enhance the durability and effectiveness of Ti_3_C_2_T_x_ MXene coatings, leading to smooth and stable frictional evolution over many cycles [392], while surface functionalization with silanes or polymer encapsulation forms protective layers that significantly improve their resistance to oxidation and prolong their lifespan in aqueous environments [394].

A key advantage outlined by M.L. Matias is the ability to process conductive, surface-tunable 2D materials into 3D architectures [261]. A limitation is the tendency of nanosheets to restack [397], which necessitates morphological control strategies to maintain accessible surface area and ion transport pathways [256,397]. Oxidation is another challenge, degrading conductivity and stability [268,399].

### 4.12. Surface Roughness and Porosity Control of Ionic Liquids and Hydrogels

The fabrication of triboelectric devices often leverages the unique properties of ionic liquids and hydrogels, particularly through 3D printing, to create specific surface architectures [270,282,400]. For instance, 3D printing techniques can produce thick electrodes with a 3D porous conductive framework structure, which significantly promotes the kinetic rate of ion transport and reduces electron transport distance, leading to high areal capacity [400]. In the context of TENGs, incorporating MXene nanosheets into polyvinyl alcohol hydrogels as a conductive electrode enhanced the short-circuit current and output performance [283]. Moreover, the development of bioinspired ionic liquid hydrogels containing MXene nanosheets has resulted in sensitive, self-healing, and conductive materials suitable for wearable sensors and electromagnetic interference shielding, showcasing a gauge factor of 2.16 [270].

Control over surface texture and porosity is critical for optimizing the performance of ionic liquid (IL)-based hydrogels in triboelectric applications, as shown by P. Lu et al. [282]. These materials, often referred to as ionogels, leverage the high ionic conductivity and non-volatility of ILs within a solid or semi-solid hydrogel matrix [270,271].

Various methods are employed to tune the structural features of these hydrogels. Three-dimensional printing techniques enable the fabrication of complex, predefined macro- and microscale structures with control over morphology and spatial arrangement [270,276]. As shown in K. Sheikh et al.’s study, direct-write 3D printing using ionogel inks can produce self-supporting conductive structures [270]. The composition, including the type and content of ILs and polymers, can significantly influence the resulting structure and properties [286,287,400,401]. For example, K.R. Hossain et al. outline that incorporating imidazolium ILs into polyethylene terephthalate affects its crystallization, melt flow, and surface roughness in 3D-printed objects [400]. Adjusting crosslinking density, for instance, by balancing phase stability and crosslinking, allows for the control of the polymer nanostructure templated by lyotropic liquid crystals containing ILs, leading to enhanced water uptake, as shown by A. Kotsiras et al. [401]. Incorporating specific components like vinyl ionic liquids and hydrophobic monomers can create multilayer microstructures, affecting hydrogel elasticity as mentioned in A. Kotsiras’s study [401]. Electrostatic interactions between components, including the IL and polymer backbone, contribute to hydrogel stability and structure [285,286,288].

These structural attributes, particularly porosity and the resulting ion transport pathways, directly influence ionic conductivity [271,276,283]. The conductivity of ionic hydrogels is provided by the presence of internal ions, as reported by J. Yin et al., which can be further optimized by introducing conductive additives such as carbon or metal nanomaterials [283]. For effective triboelectric charging, which relies on the migration of ions induced by mechanical motion, creating efficient ion transport channels within the hydrogel matrix is crucial [275,276]. For instance, as shown by J. Yin, introducing MXene nanosheets into a hydrogel structure created channels that facilitated positive ion transport after tribocharging, significantly improving the output performance of a triboelectric generator [283]. The relationship between crosslink density and ion mobility impacts ionic conductivity and TENG performance, requiring a minimum IL density for a complete charge channel, as shown by Y. Wu et al. [290]. Piezoelectric ionic skins, which convert pressure into ionic currents, also rely on differential ion mobility through the hydrogel, as shown by Y. Wang et al. [277].

Challenges include ensuring sufficient spacing between polymer chains for ion conductivity in densely crosslinked networks and preventing IL leakage with certain fabrication methods [271,276]. Consequently, as highlighted also by Y. Wu, achieving high conductivity comparable to flexible metal electrodes may also require further optimization [274,290].

Ionic liquid hydrogels and ionogels are developed to overcome stability challenges in physiological conditions, such as water loss and mechanical stress, through their inherent fluid retention and robust mechanical properties [270,283,402]. For example, lignin-reinforced poly(ionic liquid) hydrogels demonstrate impressive cyclic stability over 100 consecutive stretching–releasing cycles at 20% strain and resistance to local stress, with strategies such as incorporating ILs directly into the polymer network or encapsulation mitigating leakage and water loss to ensure longevity [270,283].

### 4.13. Surface Roughness and Porosity Control of PEDOT:PSS

Controlling the surface morphology, roughness, and porosity of PEDOT:PSS is important for optimizing its performance in additively manufactured biocompatible devices, particularly those leveraging triboelectric principles where surface interaction and charge transport are important. As mentioned before, PEDOT:PSS is widely used in 3D printing and considered biocompatible [298,299,403].

The fabrication of hierarchical and gradient surface textures in PEDOT:PSS films can significantly enhance their triboelectric performance and stability. Femtosecond laser irradiation, for example, can create hierarchical micro- and nanostructures on substrates such as platinum–iridium, providing abundant mechanical anchor points that greatly improve the adhesion and electrochemical stability of PEDOT:PSS coatings during repeated electrochemical cycling [404]. Furthermore, DIW allows for the creation of PEDOT:PSS epoxy (EP) coatings where higher PEDOT:PSS content results in smoother, more uniform, and hydrophilic surfaces with high electrical conductivity, impacting overall performance for applications like antistatic and electromagnetic shielding [311]. Such tailored surface architectures and enhanced conductivity contribute to improved charge density and overall energy conversion efficiency in triboelectric devices [273,403].

Surface roughness can be modulated through various techniques. The addition of alcohols like ethylene glycol (EG) or ethanol to PEDOT:PSS dispersions can influence film uniformity. EG can enhance triboelectric performance by significantly increasing electrical conductivity and charge transfer capacity, as demonstrated by studies showing conductivity boosts from 1.2 S/cm to 830 S/cm (F. Alhashmi Alamer et al.), with optimal EG concentrations [296,315,405]. This enhancement, according to the literature, is attributed to EG inducing a structural rearrangement of PEDOT:PSS into crystallized nanofibrils, leading to an enlarged electrical activation area and improved charge carrier mobility [296,315,405]. Such modifications also improve film roughness on the millimeter scale and enable the high-precision printing of flexible conductive patterns, with an optimized PEDOT:PSS-to-EG ratio achieving 20 µm line widths in electrohydrodynamic jet printing [298]. Furthermore, for biocompatibility, EG-enhanced PEDOT:PSS/sulfonated polyurethane (PUS) composites have shown no evidence of cytotoxicity, as reported by G. Kaur et al., making them suitable for neural implants and tissue engineering [294]. In contrast, while ethanol effectively reduces the surface tension of PEDOT:PSS dispersions, aiding uniform deposition, its addition can paradoxically decrease overall conductivity when combined with EG, likely due to a lower polymer concentration [295].

Studies such as those conducted by E. Hrehorova highlight the improving uniformity on a millimeter scale due to reduced surface tension and enhanced wetting [295]; such additives can paradoxically increase roughness at the micrometer level by inducing conformational changes and swelling of the PEDOT:PSS complex [295]. Additives such as SnO_2_ nanoparticles can also increase surface roughness in composites, as shown by A.M. Díez-Pascual [313]. Post-treatments mentioned by C. Zhang et al., such as femtosecond laser irradiation, have been shown to increase surface roughness by selectively removing the insulative PSS layer [406]. Femtosecond laser irradiation, for instance, significantly enhances electrical conductivity (e.g., from 1.2 S cm^−1^ to 803.1 S cm^−1^, as reported by L. Zhu et al.) and increases the surface roughness, which is crucial for efficient charge generation and transfer in TENGs by enlarging the effective contact area and improving work function [303,406]. Such laser-induced micro-nanostructures can also robustly enhance PEDOT:PSS adhesion and stability on critical biomedical substrates like platinum–iridium for neural electrodes, as highlighted by L. Li et al., contributing to device durability in biological environments [404].

Printing methods themselves dictate surface features; electrohydrodynamic (EHD) jet printing of PEDOT:PSS inks can achieve fine line widths ranging from 20 µm to 90 µm, influencing the resulting printed surface topography, as shown by K. Li et al. and N. Lv et al. [298,311]. Drop casting, as shown by S. Guzzo et al., tends to yield poorly ordered and highly rough surfaces, featuring nanograins (ca. 30–50 nm) [299], while spin coating can result in smoother films [406]. Roughness values vary significantly, with RMS roughness ranging from a few nanometers on flat samples to potentially much higher depending on the method and additives used [295,299,314,407]. For instance, in E. Hrehorova’s research, RMS roughness was reduced from 902 nm to 67 nm by adding ethylene glycol and ethanol to PEDOT:PSS, though AFM scans at a smaller scale showed increased roughness [295].

Porosity can be introduced using template-free methods like electro-polymerization or electrospinning, or by incorporating templates or spraying onto porous substrates, as shown in R. Brendgen et al.’s study [301]. Porous electrode designs significantly increase the surface area density, enhancing electrolyte-accessible areas for electrochemical reactions and facilitating mass transport and charge collection, as shown by A.C. Chandran et al. [408]. These structures are fundamental for enhancing charge storage and ion transport in PEDOT: PSS-based TENGs, as they promote efficient ion diffusion and accelerate charge injection and migration through expanded electrolyte accessible pathways [408,409]. For instance, PEDOT:PSS coatings on 3D-printed cellular electrodes enhanced capacitance and mitigated voltage drop, demonstrating improved ion interaction and mass loading [408]. While excessive porosity could potentially decrease overall electrical conductivity in some applications by affecting morphology [410], well-designed hierarchical porous networks can conversely significantly boost electronic conductivity and mechanical robustness, preventing conductive filler aggregation [411,412].

Surface roughness influences the real contact area during interaction as outlined by M. Bianchi et al., affecting charge transfer efficiency. The negatively charged surface of PEDOT:PSS, due to the PSS component, also influences interaction with other materials [314]. Moreover, conductivity, which is critical for collecting or dissipating generated charge, is significantly enhanced by additives such as polar solvents (e.g., DMSO and ethylene glycol), which can also alter morphology [295,296,297,304,311,315]. High electrical conductivity (e.g., up to ~62.2 S/cm in some composites—C. Gao et al. [411], or 41.50 ± 3.26 S cm^−1^ for 90% PEDOT:PSS in EP composites—N. Lv et al. [311]) allows efficient charge transport.

A.C. Chandran reports that benefits include enhanced interface interaction (relevant for charge transfer during contact) and improved charge transport through increased conductivity and surface area (in porous structures) [408]. Limitations include potential trade-offs between desired morphology and other properties, like conductivity or mechanical stability [296,299], and challenges in achieving uniform coatings and good adhesion, especially on smooth substrates [404,408,413].

Beyond traditional surface modification techniques, insights from related AM functional materials offer valuable strategies for PEDOT:PSS. Yousefi et al. showed that incorporating Fe_3_O_4_ nanoparticles into a biodegradable PLA–polybutylene adipate-co-terephthalate (PBAT) matrix via 4D printing significantly altered surface morphology and mechanical properties [412]. An optimal 10 wt% loading improved tensile strength and toughness due to enhanced interfacial bonding, while higher concentrations led to rough, flawed surfaces from nanoparticle agglomeration [412]. These nanocomposites also exhibited magneto-responsive shape memory behavior. Yousefi et al.’s work suggests that precise nanoparticle control in AM polymers can tune surface roughness and functionality insights that could guide similar strategies in PEDOT:PSS-based biomedical TENGs to enhance charge transfer, flexibility, and biocompatibility [412].

While surface morphology and porosity are crucial for optimizing PEDOT:PSS-based TENGs, significant real-world biomedical challenges persist. Long-term in vivo stability is jeopardized by the complex physiological environment, where continuous exposure to biological fluids and mechanical stress can lead to structural degradation and detachment of the polymer coating, even on engineered rough or porous surfaces designed for enhanced adhesion [403,404,414]. Maintaining robust interfacial contact with dynamic soft tissues, critical for consistent triboelectric output, remains challenging as changes in surface morphology in vivo can subtly alter protein adsorption and cell behavior, potentially eliciting undesirable immune responses [404,415,416]. Hence, translating the benefits of controlled roughness and porosity from laboratory settings to clinically viable TENGs critically depends on achieving consistent and reproducible fabrication, ensuring device longevity and predictable bio-integration [298,311,417].

PEDOT:PSS films can experience degradation and reduced performance under physiological conditions due to fluid exposure (e.g., de-doping in saline solutions [418] or swelling from moisture [419,420]) and mechanical stress, which causes cracks or delamination [404,421]. However, long-term stability is significantly improved through strategies such as femtosecond laser irradiation, enhancing adhesion and electrochemical stability on Pt-Ir substrates for over 2000 cyclic voltammetry (CV) cycles [404], and incorporating additives like natural rubber latex (NRL), demonstrating stable device operation even after 100 repetitive bending cycles for OECTs [421].

### 4.14. Comparative Assessment of Material Selection for Biomedical TENGs

The materials reviewed offer diverse capabilities for TENGs in biomedical applications, with their suitability heavily dependent on a balance of triboelectric efficiency, surface engineerability, AM compatibility, and bio-stability.

PDMS is highly versatile in surface modification (via lithography, DIW, plasma) to enhance triboelectric output, demonstrating significant voltage increases. Ecoflex^®^ offers excellent mechanical compliance, matching human tissue, and is often processed using 3D-printed molds for casting to achieve the desired porosity and output. Both show good to excellent stability under physiological and mechanical stress conditions.

Carbon-based materials, including graphene, CNTs, and rGO, offer high electrical conductivity and are highly compatible with various AM techniques. Graphene, especially LIG, can generate high power outputs and is amenable to 3D porous structures. CNTs, when dispersed effectively, can significantly enhance output, and rGO acts as an excellent electron-trapping site [422]. Nonetheless, the primary challenge for these materials in biomedical applications lies in their variable biocompatibility and potential cytotoxicity, often requiring extensive functionalization to mitigate risks and ensure long-term stability in vivo. CB, while cost-effective and AM-compatible for conductive composites, faces significant concerns regarding its biocompatibility, making it less ideal for direct bio-integration without further research into its safety profile.

Kapton^®^ and PET are well-established polymers, but their intrinsic triboelectric performance may be limited, necessitating extensive surface modification for enhanced output. While Kapton^®^ is considered biocompatible, its native triboelectric output is low. PET, despite its mechanical strength, inherently suffers from poor wettability and thrombogenicity, requiring significant modification to be biocompatible for implants. Both can be integrated with AM-related patterning techniques.

Emerging hybrid materials like MXenes and ionic liquids or hydrogels present exciting opportunities. MXenes offer remarkably high electrical conductivity and tunable surface chemistry, making them promising for high-performance TENGs, with strong AM compatibility for complex structures. Through oxidation, stability is a challenge, but it can be managed through surface modifications. Ionic liquids and hydrogels are highly flexible, conductive, and exhibit exceptional biocompatibility, making them ideal for soft, wearable interfaces, and they are readily amenable to 3D printing. PEDOT:PSS is a highly conductive polymer with favorable biocompatibility and broad AM compatibility. Its conductivity and triboelectric performance can be significantly boosted through surface engineering (e.g., laser irradiation and additives). However, its long-term stability in vivo, especially against fluid exposure and mechanical degradation, remains a critical area for improvement.

PDMS offers an excellent balance of high biocompatibility, inherent flexibility, and well-established AM compatibility. Its versatile surface modifiability allows for the significant enhancement of triboelectric output and ensures reliable performance under physiological conditions.

Due to its outstanding mechanical flexibility that closely mimics human skin and high biocompatibility, Ecoflex^®^ is an ideal choice for direct contact applications. Its proven durability under strain and good stability make it suitable for long-term wearable and soft implantable devices, often integrated via 3D-printed molds for complex structures.

Representing an advanced and promising class, MXenes offer exceptionally high electrical conductivity and tunable surface chemistry, directly translating to high triboelectric performance. Their strong compatibility with AM (Table 16) enables the creation of sophisticated, high-output structures, with ongoing research effectively addressing biocompatibility and oxidation stability challenges for future implantable and high-performance wearable applications.

## 5. Discussion

This comprehensive review has synthesized recent advancements in advanced biocompatible materials and AM techniques for the fabrication of TENGs, specifically focusing on their implications for biomedical applications. The primary objective was to elucidate the intricate relationship between material properties, surface characteristics, AM processes, and the resulting triboelectric performance within the context of developing self-powered biomedical devices for different medical areas. The findings underscore that the efficacy of triboelectric charge generation is not solely an inherent material property but is profoundly influenced by the precise control of surface morphology that includes roughness, texture, porosity, and chemical composition.

The materials examined, spanning synthetic polymers (PDMS, PTFE, Ecoflex^®^, Kapton^®^, nylon, PET), carbon-based materials (graphene, CNTs, rGO, carbon black), and emerging hybrids (MXenes, ionic liquids, hydrogels, PEDOT:PSS), present a diverse palette for TENG design. Polydimethylsiloxane stands out due to its excellent flexibility, processability, and tunable surface properties through various modification techniques, making it an effective triboelectric polymer frequently employed in TENGs. Its compatibility with AM techniques like design templating and direct ink writing allows for the creation of complex microfluidic-like structures and porous scaffolds crucial for optimizing triboelectric performance. Still, achieving optimal triboelectric output often necessitates surface modifications to enhance charge generation beyond its basic properties.

Polytetrafluoroethylene is widely recognized for its strong negative triboelectric polarity, excellent dielectric properties, and high charge storage stability, which are highly advantageous for TENG applications. While challenging to process with some AM methods due to its high melting point and viscosity, techniques like 3D printing and electrospinning (often with carrier polymers or blends) have been explored to incorporate PTFE into structured TENGs, including biomimetic designs. Its low friction and chemical inertness also contribute to its suitability, although wear resistance can be a practical limitation.

Materials like Ecoflex^®^ and Kapton^®^ offer varying degrees of flexibility and dielectric properties suitable for wearable applications. Ecoflex^®^ is noted for its elasticity and biocompatibility for wearable sensors, though its inherent insulating nature often requires conductive fillers or surface modifications. Kapton^®^ is a robust dielectric polymer commonly used as a triboelectric layer with a strong negative polarity. Although the direct AM of Kapton^®^ film for texturing is not explicitly detailed in the literature, the feasibility of 3D printing polyimides suggests potential routes for creating textured Kapton^®^ structures or composites using AM methods.

Nylon and polyethylene terephthalate are established polymers in biomedical devices with good mechanical properties. Nylon’s positive triboelectric properties and compatibility with AM techniques make it appropriate for the additive manufacturing of biocompatible systems for energy harvesting. Surface quality and roughness are critical for AM nylon parts and impact triboelectric performance, suggesting a need for control over surface chemistry and charge distribution. PET’s widespread use and potential for recycling into nanofibrous webs or aerogels for TENGs are notable. Nevertheless, its tribocharging behavior and output are significantly influenced by surface characteristics, necessitating surface modification techniques. While PET is established in biomedical devices, poor biocompatibility and thrombogenicity of some forms or modifications require careful consideration and surface treatments.

The incorporation of carbon-based materials such as graphene, CNTs, rGOs, and CB introduces enhanced electrical conductivity and mechanical reinforcement. Although their conductivity can aid charge transfer, it can also reduce the charge accumulation necessary for the triboelectric effect; hence, controlled integration and functionalization are very important. CNTs offer exceptional properties for flexible and robust components, with high surface areas influencing charge exchange. Still, challenges regarding agglomeration, dispersion within composites, and the potential toxicity of pristine forms necessitate rigorous purification and functionalization strategies for biomedical applications. rGO also exhibits inherent tribological activity and can be integrated into AM processes to develop functional composites.

Emerging hybrid materials like MXenes and ILs/hydrogels offer unique properties. MXenes, with their tunable surface functional groups and high electronegativity, are promising as friction or electrode layers in TENGs and are compatible with AM for complex structures. Their large surface area is advantageous for triboelectric performance. Ionogels represent soft ionic materials suitable for TENGs, offering conductivity and flexibility, and can be structured using direct writing and 3D printing.

A critical assessment reveals that surface texture optimization is paramount for maximizing the transferred charge density in TENGs. Micro/nanoscale structures significantly enhance the effective contact area, a decisive factor in triboelectric output. AM capabilities are indispensable in this regard, enabling the fabrication of complex geometries, specific textures, and controlled porosity that are difficult to achieve with conventional methods. Different AM methods, such as FDM, SLS, DIW, and DLP, yield distinct surface characteristics, necessitating careful process selection and potentially post-processing techniques like laser polishing or plasma treatment to fine-tune surface roughness and properties. The ability of AM to create patient-specific geometries also enables the anatomical personalization of biomedical devices integrated with TENGs.

This study synthesizes how material science, surface engineering, and additive manufacturing intersect to advance the field of biomedical energy harvesting. By reviewing the properties and AM compatibility of various biocompatible materials, the review highlights the current landscape and potential synergies for designing self-powered devices. It underscores that a comprehensive approach considering the material selection, surface design, and fabrication method is essential for optimizing TENG performance in biological environments.

Looking forward, the integration of AM into TENG research and biomedical deployment over the next decade holds immense promise but also significant challenges.

- Emerging AM-Compatible Biomaterials: Future research will likely focus on developing novel biocompatible inks and filaments specifically designed for AM, capable of incorporating advanced functionalities. This includes exploring more sophisticated hybrid materials that combine tailored mechanical, electrical, and triboelectric properties with enhanced biocompatibility and bioresorbability. The functionalization of materials during or immediately after the AM process could become standard to achieve desired surface chemistry for specific biological interactions.
- Challenges: The scalability remains a hurdle for complex AM processes, requiring industrial translation from laboratory-scale fabrication. Establishing robust regulatory standards for AM-fabricated, implantable, or long-term wearable TENGs will be critical but challenging, involving stringent requirements for material safety, device performance, and manufacturing reproducibility. Perhaps the most significant challenge is ensuring long-term biocompatibility and reliable performance under dynamic physiological conditions. Devices must withstand mechanical stress, moisture, enzymatic degradation, and the body’s immune response without losing function or causing adverse effects. Materials like CNTs and PET highlight the need to address inherent toxicity or thrombogenicity issues through functionalization and controlled fabrication.
- Future Research Directions: AM offers transformative potential for fabricating the next generation, personalized, self-powered biomedical devices; several research gaps currently limit widespread deployment, necessitating focused future directions. One of the main challenges that remains is the lack of new multifunctional biocompatible inks and filaments that can effectively overcome inherent material limitations, such as toxicity (e.g., with CNTs and PET), agglomeration (e.g., in carbon-based materials), oxidation susceptibility (e.g., in MXenes), and insufficient intrinsic mechanical robustness (e.g., PDMS or PEDOT:PSS) under physiological conditions. In addition, scalability for complex AM processes and the absence of robust regulatory standards for implantable or long-term wearable TENGs remain substantial manufacturing and commercialization hurdles. From a performance validation standpoint, a significant deficiency is the lack of systematic studies on the tribological safety of textured surfaces under simulated physiological conditions, compounded by an indispensable need for long-term in vivo studies to validate overall biocompatibility and device longevity under dynamic physiological conditions (including resistance to mechanical stress, moisture, enzymatic degradation, and the body’s immune response). Addressing these gaps will require more accurate theoretical models predicting triboelectric performance for complex AM-generated morphologies, alongside the development of advanced multi-material, multilayered AM techniques that enable the seamless integration of TENGs with other energy-harvesting modalities or sensing functions for truly multifunctional biomedical devices.
- Strategic Roadmap for Future Development: Future research should prioritize three major areas: enhancing biocompatibility through bioresorbable and AM-compatible materials, improving scalability by refining multi-material printing processes for reproducible, high-resolution fabrication, and enabling multifunctional integration by combining TENGs with sensing, actuation, or therapeutic modules.

To sum up, AM offers transformative potential for fabricating next-generation, personalized, self-powered biomedical devices based on advanced biocompatible materials. Realizing this potential necessitates continued, interdisciplinary research into material science, surface engineering, AM process optimization, and rigorous biological evaluation to overcome current limitations and pave the way for clinical translation.

## 6. Conclusions

This review has systematically analyzed recent advancements concerning advanced biocompatible materials and their integration into TENGs, specifically highlighting their relevance for biomedical applications. This study’s primary aim was to interpret findings on material performance, biocompatibility, and the essential role of surface texture in optimizing triboelectric charge generation. We summarized key materials, including synthetic polymers, carbon-based materials, and emerging hybrids, including MXenes and ionic liquid-based hydrogels. These materials were shown to possess essential properties for TENG construction, with their triboelectric performance strongly influenced by precisely controlled surface morphology and chemical composition.

The findings underscore the significant potential of these material systems for developing self-powered biomedical devices, including wearable and implantable sensors for health monitoring and energy harvesting in dynamic physiological environments because achieving reliable operation necessitates rigorous requirements for biocompatibility and performance stability. Moreover, this study reiterated the transformative impact of AM techniques, such as DIW, DLP and FDM, in enabling the fabrication of complex structures with tailored surface textures and geometries. This capability is mandatory for enhancing the effective contact area and therefore maximizing transferred charge density in TENGs.

Major future research directions include the development of new multifunctional biocompatible inks and filaments that overcome inherent material limitations such as toxicity, agglomeration, and susceptibility to oxidation through advanced functionalization and custom material design. In addition, strict in situ characterization of charge generation at additive manufacturing interfaces under physiological-like conditions and long-term in vivo studies needs to be performed to determine device lifespan and consistent performance. Novel multi-material, multilayered additive manufacturing techniques with highly controlled interfaces, along with the integration of sensing functionality and other energy-harvesting modalities, will be critical to realizing highly efficient and fully multifunctional bio-integrated devices.

## Figures and Tables

**Table 1:** Engineering methods and application potential of PDMS material in TENGs.

Fabrication Strategy	Surface Engineering	Functional Performance	Biomedical Applications	Ref.
Direct Ink Writing, DIW	ZnSnO_3_ nanocubes (6 wt%)	Max output: 400 V, 28 µA, 7 µA/cm^2^; 3 mW @ 20 MΩ; 6.2× higher than pure PDMS; stable over 500 cycles.	Flexible sensors; biological devices; Light-Emitting Diodes, LEDs.	[18]
	Carbon fiber (4 wt%)	Tensile modulus increased by 52.4% compared to pure PDMS.	Generally, for composites and 3D models.	[47]
	Porous structures (using DBP removal and fumed silica)	In total, +378% stiffness, +267% strength, +14% ductility vs. nonporous PDMS; 75% infill = best performance.	Soft robotics; biomedical devices; designing adaptive soft robots/actuators.	[43]
DLP	Thiourea groups (1.1–7.2%)	Max elongation: 1000%; excellent cyclic compression durability.	Medical devices; wearable devices; soft robotics.	[44]
Sacrificial FDM Mold	PDMS porous scaffolds with minimal surface design	Reversible under 40% strain; cell viability > 90% after 4 days (3D culture).	Scaffolding for soft tissue engineering; biocompatible porous organ-shaped scaffolds.	[43]

**Table 2:** Engineering methods and application potential of PTFE material in TENGs.

Fabrication Strategy	Surface Engineering	Functional Performance	Biomedical Applications	Ref.
DIW	PTFE micropowder for improved rheology and enhanced electron affinity	Elongation at break: 483%; low strain loss after 1000 cycles; excellent elasticity and shape stability.	Biomedical devices; soft robotics; stretchable wearable devices.	[64]
Vat Photopolymerization, VPP/DLP 3D Printing	Hydrophobic microstructures (striped and cylindrical) on PTFE; 3D-printed micro-ball and micro-mushroom arrays	Tensile strength: 22.04 MPa; yield strength: 12.57 MPa; WCA: 130.6°–132.8° (vs. 99.8° for unmodified PTFE).	Microfluidic containers; biocompatible implants; energy-harvesting TENGs.	[54,58]

**Table 3:** Engineering methods and application potential of Ecoflex^®^ material in TENGs.

Fabrication Strategy	Surface Engineering	Functional Performance	Biomedical Applications	Ref.
Molding/casting with 3D-printed mold	Prismatic mold for Ecoflex^®^/Carbon Nano-Onions, CNO composite	N/A, used as a negative electrode material for TENG.	General TENG applications.	[80]
	Polylactic Acid, PLA mold for pristine Ecoflex^®^	Output voltage: up to 17 V with Cu; similar for Al; lower electrification vs. PTFE/PDMS due to lower electron affinity.	General TENG applications and for comparative studies.	[81]

**Table 4:** Engineering methods and application potential of Kapton^®^ material in TENGs.

Fabrication Strategy	Surface Engineering	Functional Performance	Biomedical Applications	Ref.
Commercial Kapton^®^ film; 3D-printed PLA (20% infill) substrate via Sigma R19 BCN3D	Unmodified Kapton^®^ (negative triboelectric layer) paired with Mica (positive).	Mica: Kapton^®^ at 5 MΩ load: 50.1 V, 10.2 µA, 0.5 mW.	Energy harvesting for low-power electronics: LED lighting, capacitor charging.	[110]
Commercial Kapton^®^ film (not AM)	Kapton^®^ paired with rGO-modified polyimide or Ti_3_C_2_T_x_ MXene-hydrogel systems.	PI: rGO/PI: 190 V, 630 µW/cm^2^; MXene hydrogel: enhanced output (N/A).	Wearable electronics; handwriting recognition.	[5]
CO_2_ laser-structured Kapton^®^ foil	Laser-induced graphene (LIG) interdigital porous electrodes on Kapton.	Not TENG specific but produced flexible MSCs: 1.75 mF/cm^2^ (areal capacitance), 0.256 µWh/cm^2^ (energy density), 0.11 mW/cm^2^ (power density).	Energy storage in flexible micro-supercapacitors; potential in textiles, biomedical, and food systems.	[111,112,113]

**Table 5:** Engineering methods and application potential of nylon material in TENGs.

Fabrication Strategy	Surface Engineering	Functional Performance	Biomedical Applications	Ref.
Commercial PA66 film; 3D-printed PLA (20% infill) substrates using Sigma R19 BCN3D	Unmodified PA66 paired with PP or SEBS	At 5 MΩ load: PA66:PP—172.5 V, 34.4 µA, 5.9 mW; PA66:SEBS—68.1 V, 13.2 µA, 0.9 mW.	Low-power energy harvesting (LED lighting and capacitor charging).	[110]
Electrospun Nylon 66 nanofibers	Nanostructured PTFE (nanopillars) as a tribopositive pair	Voltage ↑ 6×; current ↑ 700%; charge ↑ 500% vs. unstructured pairs; stable with only 2.78% drop after 24,000 cycles.	Biomechanical harvesting (keyboard typing); wearable and sensing systems.	[125]

**Table 6:** Engineering methods and application potential of PET material in TENGs.

Fabrication Strategy	Surface Engineering	Functional Performance	Biomedical Applications	Ref.
Recycled PET processed via blending, melt mixing, and compression molding	Surface modified with polyhexamethylene guanidine (PHMG) to enhance antibacterial activity and tribopolarity.	Maximum charge density: 22.1 nC/cm^2^; among the highest reported for waste-derived TENGs.	Self-powered wearable and hygiene-monitoring sensors in direct/indirect human contact.	[146]
Electrospinning of recycled PET into nanofibrous aerogel structures	Formation of high-porosity, amine-rich nanofiber web.	Output: 67.7 V, 9.4 µA; power density: 5.47 W/m^2^; mechanical durability >10,000 cycles with 99% signal retention.	Lightweight wearable energy harvesters; sustainable small-scale devices.	[147,148]
Compression molding and melt mixing of unsorted plastic waste (PET, polystyrene–PS, high-density polyethylene–HDPE, polypropylene–PP)	Surface morphology altered by multi-material blending (e.g., PET bottle film and PS/HDPE/PP).	PET:B + PET (60% PET): charge density: 390 nC/m^2^; voltage: ~58 V (stable over 1000 cycles).	Green energy generation from mixed post-consumer plastic waste.	[149]

**Table 7:** Comparative overview of synthetic biocompatible polymers used in TENGs.

Material	Triboelectric Polarity	Biocompatibility	AM Compatibility	Ref.
PDMS	Strongly negative	Excellent; long-term cytocompatibility	DIW, DLP, molding, templates	[34]
PTFE	Extremely negative	Moderate (inert but not adhesive to tissue)	DIW, DLP, electrospinning	[21]
Ecoflex^®^	Slightly positive	Excellent; highly stretchable, skin-friendly	3D-printed molds (indirect)	[160,161]
Kapton^®^	Moderately negative	Good; stable and used in bioelectronics	Substrate-based only	[97,125]
Nylon	Positive	High; widely used in biomedical components	FFF and SLS	[110]
PET	Slightly negative	Moderate; enhanced through surface treatment	Electrospinning and molding	[149]

**Table 8:** Engineering methods and application potential of graphene material in TENGs.

Fabrication Strategy	Surface Engineering	Functional Performance	Biomedical Applications	Reference
AM (3D BioPlotter)	Graphene scaffolds (20–60 vol%) forming a continuous conductive network	Conductivity: ↑ from 200 to 600 S/m (20–60% graphene); strain range: ↓ from 210% to 81%; resistance ↑ with tensile strain; irreversible after large bending.	Nerve conduits; support for hMSC proliferation and neuronal induction.	[165,170]
AM (FFF)	PLA–graphene composites using HDPlas^®^ functionalized graphene nanoplatelets (0.3–5 µm) for improved bonding	Tensile and flexural strength: ~1.5–1.7× vs. PLA; shear strength: ~1.2× vs. PLA; slightly lower impact resistance than unfilled PLA.	Load-bearing biomedical scaffolds (e.g., bone tissue engineering).	[171]
Electrospinning	PVDF–graphene nanofibers (0–0.25 wt%) with optimized perovskite-based tribopositive layer	Power density: 11.23 W/m^2^; VOC: 245 V; ISC: 24 μA; QSC: 80.2 nC; durable: stable after 12,000 cycles and 2 months’ storage.	Energy harvesting; self-powered LEDs, capacitors, digital tubes, photodetectors.	[172]

**Table 10:** Engineering methods and application potential of rGO material in TENGs.

Fabrication Strategy	Surface Engineering	Functional Performance	Biomedical Applications	Reference
PI (Kapton)/PI:rGO/PI multilayer	rGO as an electron trap in PI; facilitates electron transfer and reduces the energy gap (confirmed by red shift in absorption).	Voltage: 190 V; power density: 6.3 W/m^2^; stable output across frequencies due to consistent charge retention.	Energy harvesting; self-powered sensing (no specific biomedical application noted).	[5]

**Table 11:** Engineering methods and application potential of CB material in TENGs.

Fabrication Strategy	Surface Engineering	Functional Performance	Biomedical Applications	Reference
Multilayered fiber TENG on PET via coating (PDMS:CB, ZnO, PVDF)	CB dispersed in PDMS coated on PET fiber; followed by ZnO and PVDF layering	Under 10 g load: ~5.1 V, ~92.5 nA; up to 10× voltage enhancement vs. prior devices; output linked to synergistic piezo- and triboelectric effects between CB-PDMS, ZnO, and PVDF	Wearable pressure sensors for smartwatches and fitness devices	[145]
Material extrusion-based AM	CB integrated in porous PDMS matrix for charge generation	N/A; device fabricated as high-efficiency triboelectric sensor	Finger-thimble sensor for potential use in healthcare monitoring	[235]

**Table 12:** Comparative overview of carbon-based biocompatible materials used in TENGs.

Material	Triboelectric Polarity	Biocompatibility	AM Compatibility	Ref.
Graphene	Positive electron affinity	Conditional; depends on surface functionalization	DIW, electrospinning, bioplotter	[226,249,250]
CNTs	Negative	Limited; concerns exist regarding cytotoxicity	Mixed into PDMS/Ecoflex^®^ composites	[233,239]
rGO	Slightly negative	Improved vs. pristine graphene; good with hydrogel matrix	Inkjet printing, casting, hydrogel formation	[5,167,251]
CB	Neutral to slightly negative	Acceptable in low concentrations; used in elastomer blends	Dispersed into PDMS, Ecoflex^®^, thermoplastics	[149,235,239]

**Table 13:** Engineering methods and application potential of MXenes material in TENGs.

Fabrication Strategy	Surface Engineering	Functional Performance	Biomedical Applications	Reference
3D Aerogel	MXene/cellulose aerogel lightweight, flexible matrix	Voltage: 115.3 V; current: 0.78 µA; power density: 402.94 mW/m^2^.	Electromagnetic shielding, energy harvesting, and self-powered sensing.	[264]
DIW (Liquid Electrode)	Stretchable MXene electrode with high electronegativity	Voltage: 300 V; current: 5.5 µA; power density: 504 mW/m^2^.	Stretchable high-output TENG applications.	[264]

**Table 14:** Engineering methods and application potential of IL and hydrogel material in TENGs.

Fabrication Strategy	Surface Engineering	Functional Performance	Biomedical Applications	Reference
3D Bioprinting	Bio-ionic ink (BIL) photo-crosslinked in hydrogel backbone; BIL content modulates properties	Enhanced conductivity, tunable mechanics, cell adhesion, anti-fouling.	Electroconductive tissue scaffolds, bioelectronics, in situ printing on tissue.	[279]
DIW	UV-curable ionogel ink forming self-supporting structures	High transparency, mechanical strength, improved electrochemical traits.	Strain sensors, tissue engineering, drug screening.	[270]
Mixed 3D Printing	PAM–LiCl ionic hydrogel integrated with 3D-printed frictional layers	N/A; optimized for low-frequency energy harvesting.	Powering low-frequency wearable/implantable electronics.	[277]
Electrospinning	PEI-modified cellulose nanofibers (CNFs), AgNP-coated, ionic liquid-dissolved cellulose matrix	Voc: 286 V, Isc: 4 µA; load density: 13.3–85.8 µC/m^2^ depending on film type.	Wearable electronics, energy harvesting, self-powered sensors.	[280,281]

**Table 15:** Comparative overview of advanced biocompatible materials and enhancements used in TENGs.

Material	Triboelectric Polarity	Biocompatibility	AM Compatibility	Ref.
MXenes (e.g., Ti_3_C_2_Tx)	Negative	Good when embedded in hydrogel/elastomer matrix	Inkjet printing and hydrogel embedding	[264]
ILs	Tunable (depends on pairing)	Moderate to good; cytotoxicity depends on ion pair	Dip coating, casting, electrostatic spraying	[273,274,275,276,277]
Hydrogels (e.g., PVA and polyacrylic acid–PAA)	Variable (matrix-dependent)	Excellent; highly biocompatible and skin-compliant	Hydrogel casting and inkjet printing	[270,277,279]
PEDOT:PSS	Slightly positive to neutral	Good; conductive polymer used in bioelectronics	Spin coating, screen printing, inkjet printing	[293,294,301]

**Table 16:** Final comparison of key properties and limitations of AM-compatible biocompatible materials.

Material	Biocompatibility	Conductivity	Surface Modifiability	AM Compatibility	Limitations
PDMS	High	Low	High	High	Softness
PTFE	High	Low	Moderate	Moderate	Difficult Processing
Ecoflex^®^	High	Low	Moderate	Moderate	Insulating
Kapton^®^	Moderate	Low	Moderate	Moderate	Low Output
Nylon	High	Low	Moderate	High	Hydrophilicity
PET	Moderate	Low	High	High	Hydrophobicity
Graphene	Variable	High	High	Moderate	Toxicity
CNTs	Variable	High	High	Moderate	Agglomeration
rGO	Variable	Moderate	High	Moderate	Dispersion
MXene	Tunable	High	High	High	Oxidation

## Data Availability

No new data were created or analyzed in this study. Data sharing is not applicable to this article.
